# Novelty in News Search: A Longitudinal Study of the 2020 US Elections

**DOI:** 10.1177/08944393231195471

**Published:** 2023-08-14

**Authors:** Roberto Ulloa, Mykola Makhortykh, Aleksandra Urman, Juhi Kulshrestha

**Affiliations:** 1 28363 GESIS – Leibniz Institute for the Social Sciences, Koln, Germany; 2 26567 Universty of Konstanz, Konstanz, Germany; 3 27210 University of Bern, Bern, Switzerland; 4 27217 University of Zurich, Zurich, Switzerland; 5 174277 Aalto University, Espoo, Finland

## Abstract

The 2020 US elections news coverage was extensive, with new pieces of information generated rapidly. This evolving scenario presented an opportunity to study the performance of search engines in a context in which they had to quickly process information as it was published. We analyze novelty, a measurement of new items that emerge in the top news search results, to compare the coverage and visibility of different topics. Using virtual agents that simulate human web browsing behavior to collect search engine result pages, we conduct a longitudinal study of news results of five search engines collected in short bursts (every 21 minutes) from two regions (Oregon, US and Frankfurt, Germany), starting on election day and lasting until one day after the announcement of Biden as the winner. We find more new items emerging for election related queries (“joe biden,” “donald trump,” and “us elections”) compared to topical (e.g., “coronavirus”) or stable (e.g., “holocaust”) queries. We demonstrate that our method captures sudden changes in highly covered news topics as well as multiple differences across search engines and regions over time. We highlight novelty imbalances between candidate queries which affect their visibility during electoral periods, and conclude that, when it comes to news, search engines are responsible for such imbalances, either due to their algorithms or the set of news sources that they rely on.

## Introduction

The 2020 US elections were one of the most viewed events of 2020, attracting 56.9 M viewers on cable and broadcast TV at prime time alone (Nielsen, 2020). As shown by the record turn-out (Schaul et al., 2020), the stakes were high in a polarized nation (Boxell et al., 2020) whose citizens were deciding the direction of a major international power. Media outlets were ready to cover every detail that would keep their visitors engaged, reporting novel pieces of information every few minutes, if not seconds (e.g., Astor, 2020; Kommenda et al., 2020). At a proportional pace, digital intermediaries, such as search engines, frantically processed the material to show the latest and most relevant updates to their audience. To complicate matters, the news coverage of one of the candidates, Donald Trump, was extraordinarily higher than any other candidate on record (Al-Gharbi, 2020). This scenario presented an opportunity to explore the performance of search engines under an intensively mediated political campaign in which political actors competed for the spotlight (Kaid & Strömbäck, 2008). This paper reports how search engines covered the elections in terms of novelty, that is, inclusion of novel items among their top news results, which, we argue, is essential for analyzing the coverage that a topic receives by the search engine.

Earlier research has shown that success in elections depends on the attention that the media spends on candidates (Hopmann et al., 2010; Maddens et al., 2006; Reuning & Dietrich, 2019; van Erkel et al., 2020). Coverage (and visibility) has not directly been addressed in news search scholarship because of the reactivity of search engines, namely, search engines do not feature a selection of materials per se (e.g., as in a news website), but retrieve them in response to user queries. For any query, search engines return a long list of news articles, albeit in the majority of cases individuals will interact with only those at the top (Pan et al., 2007; Urman & Makhortykh, 2021). Because the relevance of news items changes over time, more relevant items can appear at the top when the individual searches again. This has consequences for the visibility of the topic, as the individual would be exposed to a more diverse set of news when the novelty is higher.

Our objective is to examine the rate at which new information is integrated into the search results of various queries, search engines, and regions. Therefore, we analyze news search by investigating the novelty of results that emerges for 9 queries: 3 related to the US elections (“joe biden,” “donald trump,” and “us elections”), 3 topical (“coronavirus,” “poland abortion,” “nagorno-karabakh conflict”) and 3 stable ones (“first world war,” “holocaust,” "virtual reality”). Data were obtained during the 2020 US presidential elections from 5 search engines (Google, Bing, DuckDuckGo, Yahoo!, and Baidu); snapshots for each query were captured every ∼21 minutes between Nov 3^rd^, 07:31 a.m. and Nov 9^th^, 06:40 a.m. Eastern Time (ET) using 240 virtual agents located in two geographical areas: Oregon (United States) and a non-US location Frankfurt (Germany). The topics and the two geographical areas were selected to demonstrate that our metric can capture the higher coverage expected for the most salient topics (US elections and topical) and for a region where the elections were held (Ohio). Our focus is to investigate the evolution of news results across four periods, defined by three key events: (a) close of all polls, (b) call of Michigan’s results, the 45^th^ state being called followed by 3 days without any calls, and (c) call of Pensylvannia’s results, the state that indicated the victory to Biden.

Following (Kulshrestha et al., 2019), we include weights corresponding to the search ranking in our novelty metric to capture the tendency of individuals to click on top results more often (Pan et al., 2007; Urman & Makhortykh, 2021), including news articles (Ulloa & Kacperski, 2022). To analyze the data, we use linear mixed models, with repeated measures that stem from our longitudinal observations. First, we present evidence that novelty is indeed higher for election related queries, as well as for the COVID-19 pandemic, but neither for localized happenings outside of the US (e.g., Poland abortion protests), nor for stable queries. Additionally, we find a consistently higher novelty for the expected region, that is, Ohio. Then, we demonstrate that the novelty for the two candidates differs across search engines and that the novelty is disproportionally high, in particular for the query "donald trump" in Bing and Oregon, replicating a pattern observed in media coverage (Al-Gharbi, 2020; Rozado, 2023). To our knowledge, this study represents a pioneering analysis of the novelty of news search results, that is, we provide insights into the rate at which information is incorporated into search engines. The fine granularity of our methodology gives us enough sensitivity to demonstrate sudden changes in highly covered news topics.

## Media Coverage and Elections

Neither voters’ positions on political issues nor the candidates’ personal traits matter if the candidate is not visible to the voter (Hopmann et al., 2010). Previous research shows that electoral success depends on the attention that the media pay to candidates (e.g., Hopmann et al., 2010; Maddens et al., 2006; Reuning & Dietrich, 2019; van Erkel et al., 2020). For example, observers have attributed Donald Trump’s victory in 2016 to the amount of news coverage he received compared with his rivals (Shafer, 2016). Although news reports are guided by journalistic norms (Hackett, 1984; Muñoz-Torres, 2012), research indicates that there are market forces that influence the gatekeeping aspect of the media (Hamilton, 2011; Patterson, 2013), and that these factors were exploited by Trump during the 2016 elections (Callum Borchers, 2016; Confessore & Yourish, 2016). Not only did the news coverage for Trump soar during the 2016 election like no other candidate on record (Al-Gharbi, 2020), but it also protracted until at least 2022, that is, years after Joe Biden succeeded him in the 2020 election (Rozado, 2023).

Last-minute broadcasts which inform viewers about elections are of particular interest for the discussion of factors affecting electoral choices (Hofstetter & Buss, 1980). Such information is relevant for late deciding voters, the numbers of which have been rising in Western democracies, including in the US (see Yarchi et al. (2021) for a list of countries). For example, on election day, 12.5% of 2016 US voters were either undecided or said they planned to vote for third-party candidates (Silver, 2017). Not surprisingly, late deciding voters sometimes determine the final outcome of elections (Box-Steffensmeier et al., 2015; Schill & Kirk, 2017; Schmitt-Beck & Partheymüller, 2012). Voters that remain undecided are considered very unpredictable (Box-Steffensmeier et al., 2015, p.; Gopoian & Hadjiharalambous, 1994); they appear more reactive to campaign coverage (Fournier et al., 2004) and less critical about the information they consume (Samuel-Azran et al., 2022).

The period that follows the elections is also a sensitive one, as the legitimacy of the process is called into question by some elites that spread rumors of fraud (Minnite, 2011). Such rumors characterized the electoral campaign of 2020 US elections (Benkler et al., 2020; Berlinski et al., 2021; Enders et al., 2021) which were also accompanied by Trump’s threats of not committing to a peaceful transfer of power (Crowley, 2020). Such claims continued after the election, including the period covered by our data collection,^1^ leading to Trump supporters storming the capitol on January 6th (CNN, 2021). Hence, the post-election period is critical because the rumors are more likely to affect populations that are dissatisfied with the outcome.

## Search Engines as Digital Intermediaries

News organizations are becoming more dependent on digital intermediaries, such as search engines and social media platforms. These intermediaries represent short-term opportunities to engage audiences, even if these opportunities might result in the loss of control over their organization professional identity (Nielsen & Ganter, 2018). The technological companies behind these intermediaries are also leveraging their role to shape political communication (Kreiss & Mcgregor, 2018), while parties and candidates try to adapt their campaigns to the new media logic (Klinger & Svensson, 2015).

We focus on search engines, as they play a gatekeeper role in the current high-choice information environments (Van Aelst et al., 2017). Individuals frequently use them to seek information (Urman & Makhortykh, 2021) and learn from the results obtained (Fisher et al., 2015; Ward, 2021). Moreover, individuals rely on search engine ranking algorithms as a measure for content relevance (Edelman, 2021; Keane et al., 2008; Schultheiß et al., 2018; Urman & Makhortykh, 2021). Consequently, search engines became one of the most used technologies of finding political information (Dutton et al., 2017), which is crucial as there is evidence of their potential to shift voting preferences of undecided voters (Epstein et al., 2017; Zweig, 2017). Notably, Donald Trump has also accused that 96% of Google results on Trump news come from national left-wing media suggesting an intentional manipulation of the US audience (Satariano et al., 2018).

Specifically, we are interested in the coverage of topics in search engines. Instead of looking at a single result page in which virtually all items presented are pertinent to the query, we look at novelty, that is, the number of novel items that emerge in the top results. We argue that higher novelty increases the visibility of the topic. First, an individual is more likely to encounter more information if they search more than once for the same topic at different points in time. Second, it increases the potential amount of information that can be circulated via the searcher’s personal network due to the effects of interpersonal communication (Katz & Lazarsfeld, 2017; Schmitt-Beck, 2003). Third, given that recency plays an important role in the ranking of results (Dong et al., 2010), there could be spillover effects to other elements of search engine interfaces (e.g., news featured in the main search results).

## Search Engine Auditing

Search engines have attracted a lot of attention in the algorithm auditing field, which investigates performance of algorithmic systems and their potential biases (Mittelstadt, 2016). First, researchers have reported a concentration of results of a few news sources for different Google interface components such as the main search results (Jiang, 2014), Google Top Stories (Kawakami et al., 2020; Trielli & Diakopoulos, 2019), news search (Nechushtai & Lewis, 2019), and video search (Urman et al., 2021a). These findings extend to the Dutch (Courtois et al., 2018) and German context (Haim et al., 2018; Unkel & Haim, 2019).

Second, Pariser (2011) argued that search personalization, that is, content selected according to previous individual’s consumption and preferences, could lead to filter bubbles, that is, feedback loops of information which hinder exposure to different views. Current empirical evidence indicates that such concerns are overstated, and that, instead, search engines can lead to an increase of diversity of news sources that are consumed (e.g., Stier et al., 2022; Ulloa & Kacperski, 2022).

Third, several aspects of political representation have been investigated. Puschmann (2019) finds that some political parties and candidates can exert greater influence over how they are represented in search media (in terms of source type) than others. There is also evidence suggesting a (modest) left partisan leaning in Google search results (Robertson et al., 2018; Trielli & Diakopoulos, 2019), although the leaning is usually measured on the source and not necessarily the content level (Ganguly et al., 2020).

Only few studies conduct longitudinal investigations: Metaxas & Pruksachatkun (2017) reported that Google (but not Yahoo! and Bing) restricted variation of sources across time, favoring those that were considered “reliable” to prevent the surfacing of “fake news.” Kawakami et al. (2020) found that a year before the US elections 2020, the number of unique news in Google’s Top Stories differed for different candidates, and it was higher for Donald Trump, which was attributed to him being the incumbent president. Pradel (2021) found gender and party differences in the amount of personal information related to politicians that appear on the search suggestions before and after the elections. Closer to our work, Metaxa et al. (2019) systematically analyzed daily search results, finding search outputs to be relatively stable, though some shifts suggested the existence of internal algorithmic factors, for example, monthly synchronization of Google servers.

Most of the works have investigated Google exclusively, however there are exceptions that demonstrate differences between search engines in terms of source concentration (Jiang, 2014), “gaming” or “link bombing” during the 2008 US Congressional Elections (Metaxas & Mustafaraj, 2009), content diversity (Steiner et al., 2020), preventing “fake news” (Metaxas & Pruksachatkun, 2017) and low results overlap between search items obtained by queries of candidates of the 2020 U.S. Presidential Primary Elections (Urman et al., 2021b).

## Research Questions and Hypothesis

Our aim is to analyze the rate at which new information is incorporated in the search results of different queries, search engines, and regions. To our knowledge, this is the first time that novelty of news search results is analyzed, that is, we give first insight into the pace at which information is integrated into the search engines. The fine granularity of our data collection (every 21 minutes per query) allows us to capture sudden changes.

We first contrast US-related queries with other topical queries—we chose COVID-19 (“coronavirus”), the Poland abortion protests following the Constitutional Tribunal ruling on October 22, 2020 (“poland abortion”) and the 2020 Nagorno-Karabakh conflict dated 27 September 2020–10 November 2020 (“nagorno-karabakh conflict”), for which we also expected relatively high coverage and novelty of news articles. Additionally, we included stable queries (“first world war,” “holocaust,” “virtual reality”), for which we expected a low amount of novel news. These categories serve as a benchmarks to demonstrate the coverage given to novel items related to the US elections, see RQ1 in Table 1.

**Table 1.** Research questions and related hypotheses of the present study. The first column identifies the research question or hypothesis presented in the second column. The third column indicates if the hypothesis is supported or not: consistently, means that almost all (or all) cases side with the hypothesis; partially, if there are notable counterexamples that need to be explained; and rejected, if most of the evidence sides with the opposite direction of the hypothesis. (*) Specifically for RQ1, from Nov 3^rd^, 07:31 a.m. to Nov 4^th^, 11:10 a.m. ET.

ID	Research question and hypotheses	Supported
RQ1	Is the novelty of queries related to the US elections higher than other queries during election day and the hours following it (*)?	
H1a	The novelty for queries related to the US elections is higher than for queries related to other topics, especially those not news-worthy during the same period (i.e., stable queries such as the First World War)	Consistently
H1b	More novelty is displayed for topical queries during the collection (e.g., “poland abortion”) than those not news-worthy (e.g., “first world war”)	Partially
RQ2	Are there differences in novelty for the different US elections related queries, regions, periods and search engines?	
H2a	More novelty is displayed in Oregon (United States) than in Frankfurt (Germany). Given the role that localization plays in search results (Kliman-Silver et al., 2015), we assume that more attention is drawn to the topic in the US.	Consistently
H2b	There are differences in the novelty of results shown by different search engines	Consistently
H2c	Specifically, Google will display less novelty than Bing and Yahoo! as previous research indicates that their organic results show less variation over time, presumably as a consequence of potential mechanisms to control web spammers (Metaxas and Pruksachatkun, 2017). We assume a similar trend for news results	Partially
H2d	Novelty of the US queries diminishes as results are more distant from election day and stories become less abundant in news	Consistently
H2e	There are no differences between novelty of the candidates in different search engines before the announced election result (periods I, II, III)	Rejected
H2f	Novelty of results for “joe biden” will be higher than for “donald trump” after the declaration of Biden as a winner (period IV)	Consistently, but not for all period IV

We further examine the evolution of the novelty for the US elections related queries. First, we divide our collection in four periods (denoted with roman numbers: I, II, III, and IV) defined by three key events: (a) close of all polls (Nov 4^th^, 1:00 a.m. ET), (b) call of Michigan’s result (Nov 4^th^, 5:58 p.m. ET), the 45^th^ state being called followed by 3 days without any calls, and (c) call of Pennsylvania’s results (Nov 7^th^, 11:25 a.m. ET), the state that gave the final victory to Biden. Then, we examine differences between periods, regions, and search engines. We pay special attention to differences between the queries of the two candidates to find imbalances in novelty. See RQ2 in Table 1.

## Materials and Methods

For our data collection, we used virtual agents, that is, software that simulates human behavior (Ulloa et al., 2021). The implementation of such an agent took the form of a browser extension (for Firefox and Chrome) that simulates the navigation of search result pages on a search engine, and that collects the HTML of the pages by sending it to a server. The agent collects at least 50 news search results (if available), and it iterates over the list of terms until terminated. Before starting the search for a new query, the browser data (e.g., history, cache) is cleaned, thus avoiding personalization effects based on previous browsing history. We parsed the HTML pages to extract the top organic news results of each search routine.

### Data collection

We used the news search engine results collected in two consecutives experiments which included 9 terms divided equally in three categories (see Table 2). A category was assigned to each agent, and each of the three terms in the category were queried sequentially in a continuous loop, so that each term was searched every 21 minutes (a search routine lasts 7 minutes per term). The data was collected from Nov 3^rd^, 07:31 a.m. to Nov 4^th^, 11:10 a.m. ET, accounting for 80 rounds (collection A). Additionally, the collection for the US category (US-related queries) was extended until Nov 9^th^, 06:40 a.m. ET (extra 329 rounds, collection B).

**Table 2.** Terms of each query category. The first column displays the name of the query category, the second column the terms included in the category, the third column the topic they are related to, and the fourth column, the experiment (s) in which they were included.

Query category	Terms	Related to	Collections
US	joe biden, donald trump, us elections	US elections 2020	A, B
Topical	coronavirus, poland abortion, nagorno-karabakh conflict	Issues that were highly covered by the news at the time	A
Stable	first world war, holocaust, virtual reality	Topics that we consider were not being of news importance at the time	A

For collection A, a total of 240 virtual agents were deployed simultaneously in the Amazon Elastic Compute Cloud (using 120 CentOS virtua l machines, each hosting two virtual agents: one in Chrome and one in Firefox), and the agents were distributed equally to each experimental condition given by the combination of variables in. In total, each experimental condition was assigned to 4 different agents, so that we could account for the effects of results’ randomizations by the search engines (Makhortykh et al., 2020). Additionally, all machines on a given region were allocated in the same range of Internet protocol (IPs). For collection B, we reduced the scale of the experiment to keep costs under our budget (as an election winner did not emerge until days after), so all machines assigned to the topical and stable categories were terminated and only 1 agent per condition was kept for the US category (20 agents in total).

In Appendix S1, we include a detailed analysis of the data collection coverage. In general, very good coverage can be reported for our analyses and although some systematic issues are reported, we make sure that our analysis are not directly affected by them. Additionally, the weighting of the ranking, presented in the next section, improve on potential distortions.

### Definitions and Metrics

#### Item

It describes the combination of a URL and a title in a news search result. An item is the main unit of analysis in this paper because some URLs are used as live streams (e.g., https://www.nytimes.com/live/2020/11/07/us/biden-trump) to dynamically publish different pieces of information. Thus, the URL does not uniquely identify a news search result.

#### New Items

We define that an item in round *j* is new if it is the first time that it appears for a given query term and virtual agent; conversely, an item is not new if it appears in any previous round *i* (i.e., *i* < *j*) for that term and agent. The following items are discarded as we cannot ascertain if they are new or not: (1) items that appear on the first (successful) collection round, (2) items of a round *j* that follows a missing or incomplete round *j-1*.

#### Weighted Rank

All our metrics (except diversity) consider the position (rank) of the search results. For this, we generalize the weights used to estimate the (political) biases on search results (Kulshrestha et al., 2019). In their work, each rank in the list is assigned a weight such that higher weights are assigned to higher ranked results (i.e., top results), which is then multiplied by the (political) bias score of the corresponding item. Let *L* be the sequence of items (*i*) of size *N* corresponding to the top results of a query in a given round, the weight for the rank *r* is calculated as follows:

$$W\left(r,N\right)=\frac{1}{N}\;\overset{N}{\underset{c=r}{\sum }}\frac{1}{c}$$

#### Novelty

We define a parameter δ that takes the values 1 or 0 ( δ ) depending on whether the item is new or not, and use the weighted rank measure to calculate the novelty of the sequence *L*:

$$Novelty\left(L\right)=\overset{\left|L\right|}{\underset{i=1}{\sum }}\delta_{i}\cdot W^{\prime }\left(i,\left|L\right|\right)$$

where $W^{\prime }$ is a re-scaled weight that accounts for missing items, otherwise they would be implicitly counted as zeros, that is, not new news items. We assume that missing items of an incomplete round should occur independently, therefore counting them as 0 would bias the calculation (decreasing the novelty). Let $L^{\prime }$ be the set of collected items, then the weights are re-scaled as follows:

$$W^{'}\left(r,N\right)=\frac{W\left(r,N\right)}{\sum_{i=1,l_{i}\in L'}^{N}W\left(i,N\right)}$$

Note that the novelty ranges from 0 to 1. To give an intuitive idea, a new item that appears of the top position (out of 50) represents a novelty of .089 (i.e., 8.9% change of the information assuming that the weights are an adequate way of representing the relevance of the results), whereas a change on the 10th represents a novelty of 3.3%. The novelty of a change in both, the 1st and 10th, results is represented by the addition of the two novelties, that is, 12.3%.

### Study Design and Analysis

Our study considers several factors that affect the search results: search engine, region, query (or query category) and period. The first three are described in Tables 2 and 3. We define four periods according to three key events (close of all polls, call of Michigan’s result and call of Pennsylvania’s results). As an independent (continuous) variable, we analyze novelty as described before.

**Table 3.** Variables of the Experiment. The first Column Displays the Name of the Factor, the second Column the Possible Values of each Factor, and the third Column the Number of Values in each Variable.

Variable	Values	N
Region	Oregon, Frankfurt	2
Browser	Chrome, Firefox	2
Search engines	Baidu, Bing, DuckDuckGo, Google, Yahoo!	5
Query categories	US elections, topical, stable	3

To answer the research questions (Table 1), we used linear mixed-effect models (Bates et al., 2015), fitting the interaction between the study factors (query or query category, period, engine, and region). We considered the following random intercepts for repeated measures: query term (when query category is one of the factors), agent and round. However, we only report the models with the lowest Akaike’s Information Criterion (AIC) (Akaike, 1974); in case of models not being statistically different, we kept the simplest of them. For novelty, we tested two types of models according to RQ1 (query category, engine, and region) and RQ2 (query, engine, region, period).

To evaluate our hypotheses, we count the relevant contrasts that are significantly different and support the hypothesis (or contradict it). The contrasts are calculated on the fitted model using the emmeans R package (Lenth, 2021). All our plots include bootstrapped confidence intervals (95%); in the case of time series, rolled averages (and confidence intervals) are calculated based on the observations of the previous 6 hours.

## Results

**Figure 1.**
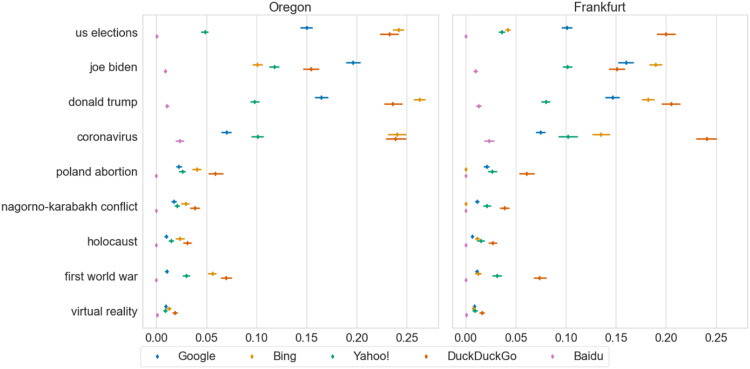
Novelty of query terms. The Y-axis shows the query terms that were explored, and the X-axis shows the novelty (truncated to .3, maximum theoretical value: 1.0). The legend shows the different search engines. The left plot corresponds to Oregon and the right plot to Frankfurt. Bootstrapped confidence intervals at 95%.

We found a triple interaction between the query category, engine, and region; F(8, 207.266) = 9.205, *p* < .001 (Appendix S4). The US-related queries displayed significantly more novelty than the topical and stable queries for Bing, DuckDuckGo, and Google in both regions (.10 < β < .23, *p* < .007) except between US- and topical-related queries for DuckDuckGo in Frankfurt. No significant differences were found between the topical and stable queries. Thus, we found support for H1a, but not for H1b. Figure 1 presents the results by query indicating that “coronavirus” is carrying the effect of the topical category. To confirm this, we fitted another model (Appendix S5) with an exclusive category for the "coronavirus" query, which was consequently removed from the topical category. In this new model, the US-related queries displayed significantly more novelty than the topical and stable queries for all regions and engines (.08 < β < .22, *p* < .001), except for Baidu (NS). Additionally, for Google, the US-related queries displayed significantly more novelty than the "coronavirus" query (−.13 < β < −.07, *p* < .001). Given the generally low novelty of Baidu, we will not consider it for the rest of the analysis.

**Figure 2.**
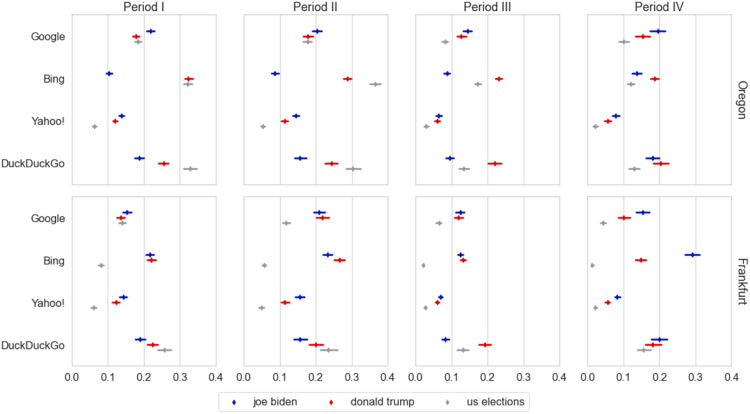
Novelty of US-related queries across periods. The X-axis shows the novelty truncated to .4 (maximum theoretical value: 1.0), and the Y-axis the engines. The legend identifies the US-related queries. The top row corresponds to Oregon and the bottom one to Frankfurt. The columns present the results per period. Confidence intervals at 95%.

To analyze the difference between the US-related queries, we fitted a model (Appendix S6) including the three queries and the four periods (Figure 2). We found a quadruple interaction; F(18, 789420.858) = 17.45, *p* < .001. To understand the patterns of this interaction we analyzed the contrasts in four steps according to our hypotheses. First, we analyzed the hypothesis that the novelty was higher for Oregon (H2a), which was supported by 9 (out of 16) contrasts for “us elections” (−.11 < β < −.03, *p* < .001), by 6 (out of 16) for “donald trump” (−.11 < β < −.03, *p* < .001) and 1 (out of 16) for “joe biden” (β = −.06, *p* < .001), and rejected by 4 (out of 16) contrasts for “joe biden” (all corresponding to Bing; .03 < β < −.15, *p* < .001), 1 (out of 16) contrast for “donald trump” (β = .037, *p* = .001) and none for the “us elections.”

Second, we found significant differences between the novelty of different search engines (H2b) as shown in Table 4. Yahoo! consistently displayed the least novelty, while DuckDuckGo always occupied the first or second position. Bing occupied the first position in 4 (out of 6) combinations of query regions but shared the last position with Yahoo! for “joe biden” in Oregon, and “us elections” in Frankfurt. Google occupied the third position 3 times, the second 2 times and the first one time. Therefore, we only find partial support for H2c: Google displayed more novelty than Yahoo! in all cases and less novelty than Bing in 4 out of 6 cases; this held true for all periods regardless of the changes observed in specific periods (see last column of the table).

**Table 4.** Engines sorted according to novelty. The first column displays the region, and the second the query. Column 3 to 6 indicates the position that the search engine took according to their novelty (in parenthesis); if there is no statistical difference between two engines, they are displayed in the same cell separated by column. The last column indicates the periods for which the order held true; the italics indicates when the order of the non-statistical differences were switched.

Region	Query	1^st^	2^nd^	3^rd^	4^th^	Periods
Oregon	Joe Biden	Google (.19)	DDG (.16)	Yahoo! (.11), Bing (.10)		I, II, *III, IV*
	Donald Trump	Bing (.26)	DDG (.23)	Google (.26)	Yahoo! (.09)	I, II, III. IV
	Us elections	Bing (.25)	DDG (.22)	Google (.14)	Yahoo! (.04)	*I*, II, III, *IV*
Frankfurt	Joe Biden	Bing (.21)	Google (.16), DDG (.16)		Yahoo! (.12)	*I*, III, *IV*
	Donald Trump	DDG (.20), Bing (.19)		Google (.14)	Yahoo! (.09)	I, III, IV
	US elections	DDG (.19)	Google (.09)	Bing (.04), Yahoo! (.04)		I, II, *III, IV*

**Figure 3.**
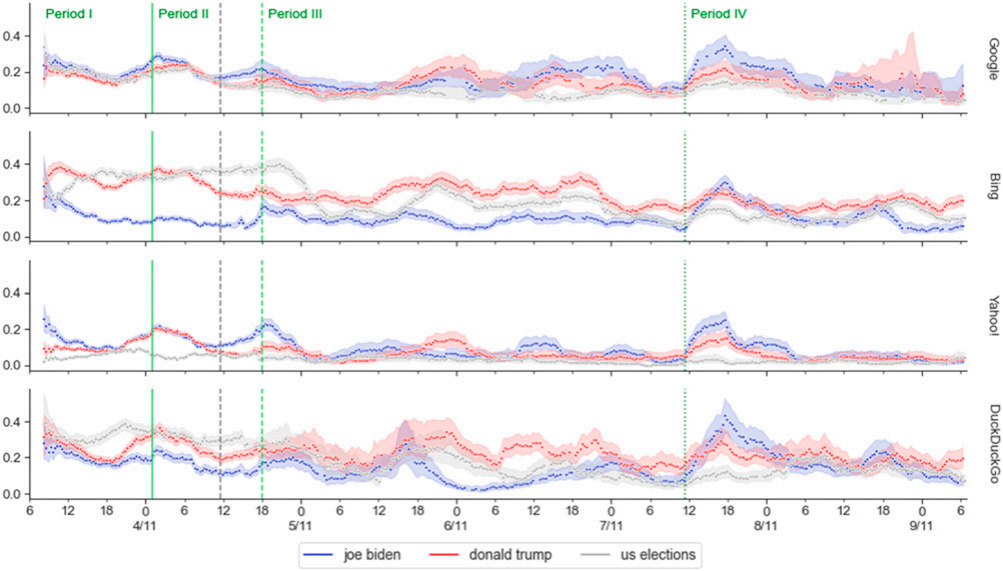
Novelty of search results over time in Oregon. The four plots present the rolled average novelty (of the last 6h, *n* = 18) for each search engine (right label). The X-axis shows the day (major ticks) and hour (minor ticks) of the round in which the novelty was measured. The Y-axis shows the novelty truncated to .4 (maximum theoretical value 1.0). Each trace represents each of the query terms indicated on the legend. The green vertical lines divide each plot in four periods indicated in the label at the top. The gray dotted vertical line in Period II indicates the transition between collection A and (b) Only the results collected in Oregon are shown. The bands indicate bootstrapped 95% confidence intervals.

Third, we analyzed the novelty of subsequent periods on all US-related queries for Oregon (H2d): 65 (out of 144) contrasts supported the hypothesized downward trend as time passed from election day (.04 < β < .21, *p* < .008). 10 contrasts contradicted the hypothesis (−.15 < β < −.04, *p* < .001), out of which, 6 involved period IV for “joe biden” which can be explained by the spike of news for “joe biden” after he was declared the winner (Period IV, Figure 3).

Fourth, we analyzed the contrasts between the candidates queries in Oregon for Period I to III (H2e). For Oregon, 8 out of 12 contrasts contradicted the hypothesis of unbiased novelty in Oregon; this included all contrasts of Bing (.14 < β < .22, *p* < .001), and DuckDuckGo (.06 < β < .13, *p* < .001), where we found more novelty for “donald trump” than for “joe biden,” and one for Google (Period I, β = −.04, *p* < .001) and Yahoo! (Period II, β = −.03, *p* < .001), in which we found the opposite. The unbalance for Bing in Oregon is particularly disproportionate: at the end of the Period III, there are 3.24 times as many unique news items for “donald trump” (*N* = 3599) as there are for “joe biden” (*N* = 1110). This is followed by DuckDuckGo, with 1.99 times as many results for “donald trump” in Oregon (and 1.77 in Frankfurt, see Appendix S7 for other proportions). For Frankfurt, the results were more balanced: only 3 out of 12 contradicted the hypothesis in the same directions, according to search engine: Bing (Period II, β =.03, *p* < .001), DuckDuckGo (Period III, β = −.11, *p* < .001), and Google (Period II, β = −.04, *p* < .001).

Fifth, we analyzed novelty displayed by the candidate queries in Period IV (H2f): 3 (out of 4) contrasts in Frankfurt supported the hypothesis that Biden’s query would display more novelty than Trump’s (−.15 < β < −.02, *p* < .003). In Oregon, only one contrast was significant but contrary to the hypothesis (β = .05, *p* < .001). Since the evidence to support H2e remained contradictory, we supported it with a time series visualization (Figure 3). The spike of novelty generated, after Pennsylvania was called (Period IV), signaling the victory of Biden, is noticeable in all search engines; at their peaks, “joe biden”’s novelty was highest in all cases (also in Frankfurt, Appendix S8), but we also noticed that its novelty quickly declined, and, at least, in Bing and DuckDuckGo, “donald trump”’s novelty increased after the spike (similar to previous values). The latter observation is consistent with the bias noted in Periods I to III (which rejected hypothesis H2e). Additionally, the spike of Biden’s novelty in Period IV was strong enough to explain the two contrasts that did not support the hypothesis of a downward trend in novelty as time passes (H2d).

## Discussion

Using the novelty of news results, we confirm that the US elections were widely covered by all search engines (H1a) except for Baidu, the only non-US search engine we included. The “coronavirus” query was the only other query that displayed similar novelty; in other cases, we do not find differences between the topical and stable queries, which highlights the tendency to neglect localized but topical and news-worthy happenings such as the Poland abortion protests and the Nagorno-Karabakh conflict (H1b).

Although we find several differences between the novelty displayed by each search engine (H2b), we only find partial support for the hypothesis that Google displays less novelty than Bing and Yahoo! (H2c), as was the case for the main search results in the US elections 2016 due to spam control mechanisms (Metaxas & Pruksachatkun, 2017). Specifically, there is partial support for Bing, but not for Yahoo!. It is possible that these search engines have now implemented spam control mechanisms similar to those of Google (thus, changing the trends from 2016).

We find support for decreasing novelty as searches become more distant from the elections period (H2d) as the great majority of significant contrasts (65 out of 75) show a downward trend. Moreover, 6 out of the remaining 10 are explained by a rebound in novelty for “joe biden” in Period IV due to the spike of news caused by his victory (Figure 3). Although the spike can be observed in all time series, it is not enough to compensate for the downward trend in all cases. A single relevant event (the victory of Biden) may not have resulted in a diverse emergence of information compared to the numerous smaller events that occurred throughout the election period. This does not imply a lack of news coverage from various sources; rather, it suggests that a few sources quickly gained prominence and remained at the top of the results, while other sources that appeared later did not have a chance to become relevant. This has direct consequences in the existing pressure for fast reporting of news and deserve future analysis could shed light on this phenomenon.

We find differences in the novelty of the results concerning the election candidates in Periods I, II and III (H2e). It differs according to the search engine, with Bing and DuckDuckGo displaying more novelty for Trump, and Google and Yahoo! for Biden. This imbalance is particularly high for Bing, resulting in 3.24 times more unique news items for Trump. Bing replicates, and potentially over-represents, the imbalances of the news coverage reported by (Al-Gharbi, 2020; Rozado, 2023) - from their visualizations, we estimate that Trump was mentioned 2.5 times more than Biden. The search engine shares responsibility for such an imbalance that might be favoring the propagation of Trump’s messages, including multiple claims of fraud (Benkler et al., 2020; Berlinski et al., 2021; Enders et al., 2021). Independently of the potential for spreading misinformation, for the period before polls closed (Period I), there are still potential undecided voters seeking last-minute information who might be exposed to a higher number of articles about Trump. The results concerning Bing wouldn't be as striking if other search engines would have not displayed such a stark bias. In two cases (Google and Yahoo!), the analysis even suggests an over-correction that produces comparatively much smaller imbalances in the opposite direction (i.e., more novelty towards Biden).

Crucially, for the period after Biden was declared the President Elect (Period IV), we predicted that there would be more novel news articles for “joe biden” (H2f), but we found the novelty to be resilient: after a spike in the novelty that favored Biden at the beginning of the period it shifted back to similar values of previous periods, for example, in Bing, the novelty favored Trump again after the spike, which is consistent with previous analysis (Rozado, 2023). Another result that sets Bing apart is that it was the only search engine which consistently displayed more novelty for Biden in Frankfurt, contradicting H2a. Such attrition of novelty could be attributed to stronger spam mechanisms in Oregon, however, that explanation would make even more puzzling the higher novelty for “donald trump” in Oregon, as it would indicate that more content was blocked for “joe biden” for no apparent reason. A more consistent explanation is that Bing was again replicating news media patterns, this time coming from German news outlets.

We find multiple differences between search engines. However, we observe some qualitative parallels between two search engine pairs in Oregon: Bing and DuckDuckGo, and Google and Yahoo!. First, Bing and DuckDuckGo showcased on average more novelty than Google and Yahoo!. Second, Bing and DuckDuckGo both displayed more novelty for Trump than for Biden, while Google and Yahoo! displayed close values of novelty for both candidates. DuckDuckGo explicitly acknowledges a relationship with Bing (DuckDuckGo, 2022) while Yahoo! has had partnerships, first with Bing (BBC, 2009) and then with Google until 2018 (Statt, 2015), though we were not able to verify if such partnerships still exist and if they extend to the news search. Shall this observation be correct, not only does Google capture 91.4% of the worldwide market, followed by Bing with 3.3% (Statcounter, 2022), but these two might influence the two here investigated alternatives, Yahoo! and DuckDuckGo, exacerbating a monopoly on the control of online information.

Aside from our findings, we present a series of methodological contributions. First, by establishing the volatility of news results during critical periods such as elections, we highlight the need for monitoring electoral processes longitudinally, where scattered snapshots might miss the big picture. Second, while other works have focused on the stability of search results (Metaxa et al., 2019), we introduced novelty, an indicator that measures how much new information is introduced. Third, we generalized the use of ranking bias used previously for political leaning (Kulshrestha et al., 2017, 2019) to our novelty measurement.

We list some limitations of our study. First, our results only cover one region in the US: Oregon. Instead of choosing two US regions, we decided to include Frankfurt as we were interested in international localization differences. Second, a list of three queries (of the same category) was assigned to each agent. Although the searches are synchronized across agents, the second query of the list is shifted 7 minutes (for each given round), and the third query, 14 minutes. Nevertheless, we argue that this should not affect the general patterns of the observed results as they are relatively small shifts. Third, we only include three queries per category. Fourth, we only analyze a small set of queries, but we point to potential spill overs that could emerge given the importance that search engines place on the recency of results. The observed imbalances could emerge (1) in other queries, for example, one could analyze if the novelty in the “us elections” is properly balanced between the candidates, and (2) in other sections of the search engines, for example, similar to the imbalances found for Google Top Stories section (Kawakami et al., 2020).

The present work also opens the door to future research regarding the visibility of candidates in news search, for example, the presence of each candidate in general queries (e.g., “us elections”) or the news results that infiltrate the main search results. As the literature indicates, not only visibility, but also tonality, is important in terms of political choices (Hopmann et al., 2010), for which natural language processing techniques could be applied (Hutto & Gilbert, 2014). Finally, it is important to study the relation between novelty and information related to fraud claims and, in general, the presence of misinformation.

## Conclusion

The existent relation between news organizations and political campaigns continues its transformation, as digital intermediaries such as search engines leverage their influence to shape political communication. We started this investigation to learn how search engines process the quick turnover of news content generated during highly mediated political events such as the 2020 US elections. We argue that our metric, novelty, allows the investigation of coverage and visibility of topics in search engines, and we demonstrate differences across search engines, regions, and periods. We find an imbalance in novelty between the candidate queries, particularly large for Bing in Oregon. Bing replicated an existent pattern in the news coverage of the candidates in the United States, while other search engines counteracted it. Contrary to the main web search, in which biases can be explained by the difficulty of balancing the enormous quantity of content available online, the number of available news articles is comparatively small and limited to a more defined set of sources that search engines already control for, and which suggests an easier target for regulation. Thus, search engines share a larger responsibility in providing a balanced coverage—either in their algorithms or in the criteria used in the selection of news sources. Such imbalances in novelty affect the visibility of political candidates in news search.

## Supplemental Material

Supplemental Material - Novelty in News Search: A Longitudinal Study of the 2020 US Elections Supplemental Material for Novelty in News Search: A Longitudinal Study of the 2020 US Elections by Ulloa Roberto, Makhortykh Mykola, Urman Aleksandra, Kulshrestha Juhi in Social Science Computer Review

## Note

1. There are 98 fraud claims related to the elections attributed to Trump in The Washington Post database during our collection period (Nov 3^rd^ to Nov 11^th^, 2020), 79 occurred after the election day https://www.washingtonpost.com/graphics/politics/trump-claims-database/.
