# Supplementary material for: Novelty in News Search: A Longitudinal Study of the 2020 US Elections
Source: Soc Sci Comput Rev. 2023 Aug 14;42(3):700–18. doi: 10.1177/08944393231195471 (PMC11093710; doi:10.1177/08944393231195471)
Supplement: Supplemental Material - Novelty in News Search: A Longitudinal Study of the 2020 US Elections [file sj-pdf-1-ssc-10.1177_08944393231195471.pdf]

Appendix S1. Data Coverage

Repeatedly collecting numerous data snapshots (every 7 mins) for a long period of time posed challenges that caused data losses. For example, virtual agents might not start properly, and there are browser and network failures. Thus, we report on data coverage to assess the quality of the data.

For experiment A, 240 agents were assigned to the different conditions, i.e., 8 per region, engine, and query category. At least 3 agents (out of 8) collected data for each condition and round (N=79). 10 agents did not initialize and 12 failed during the first round. Only 1 of those corresponded to Oregon (for the topical query category). The only seemingly systematic error involved all the Chrome agents in Frankfurt, and we discarded the possibility of a human error in the set up because 8 did collect data on the first round. The coverage achieved by the remaining machines (N=222) was high (96.05%), excluding Baidu in Frankfurt, of which the coverage was low 73.89%. More details of the coverage per condition can be found in TableS1.

		Baidu	Bing	DuckDuckGo	Google	Yahoo!
**Frankfurt**	**elections**	61.9 (4.9 / 8)	94.2 (6.6 / 7)	82.5 (5.8 / 7)	85.4 (6.8 / 8)	100.0 (4.0 / 4)
	**topical**	73.7 (5.9 / 8)	91.7 (6.4 / 7)	91.3 (7.3 / 8)	94.6 (6.6 / 7)	100.0 (4.0 / 4)
	**stable**	86.1 (6.9 / 8)	91.5 (6.4 / 7)	95.7 (7.7 / 8)	100.0 (8.0 / 8)	100.0 (4.0 / 4)
**Oregon**	**elections**	99.1 (7.9 / 8)	100.0 (8.0 / 8)	94.3 (7.5 / 8)	97.6 (7.8 / 8)	100.0 (8.0 / 8)
	**topical**	98.1 (7.8 / 8)	100.0 (7.0 / 7)	100.0 (8.0 / 8)	95.4 (7.6 / 8)	100.0 (8.0 / 8)
	**stable**	86.6 (6.9 / 8)	98.6 (7.9 / 8)	100.0 (8.0 / 8)	100.0 (8.0 / 8)	100.0 (8.0 / 8)

Table S1. Coverage of collection A. The table shows the coverage per region (first column), query category (second column) and search engine (first row). The main value of each cell shows the percentage of rounds (per agent) for which search results were collected; the first value in the parenthesis shows the average number of agents that collected data for each round, and the second value the number of agents that completed, at least, the first round.

For experiment B, 20 agents were assigned to the different conditions, i.e., 2 per region, engine, and query category. No errors of initialization were found, but 5 agents performed very poorly: 3 agents corresponding to Baidu, which we discarded for Experiment B due to lack of novelty (see Results), the Firefox agent for DuckDuckGo in Frankfurt (9.5%) and the Chrome agent for Yahoo! in Oregon (7.9%). Excluding Baidu, we collected data for more than 89% of the rounds, except for Bing in Frankfurt with only 80.5% (in which we lack data for the last 63 rounds). More details of the coverage per condition can be found in Table S2.

		Baidu	Bing	DuckDuckGo	Google	Yahoo!
**Frankfurt**	**donald trump**	14 (4.3,13.7)	80.5 (80.5,76.2)	99.7 (99.7,7.3)	95.7 (80.2,68.6)	99.4 (99.4,99.4)
	**joe biden**	14.3 (4.3,14)	80.5 (80.5,76.5)	99.7 (99.7,9.5)	97.6 (79.3,72)	99.7 (99.7,99.7)
	**us elections**	14.3 (4.6,14)	80.8 (80.8,76.8)	100 (100,9.5)	94.8 (77.4,63.7)	100 (100,100)
**Oregon**	**donald trump**	89 (0.9,89)	99.7 (99.7,99.7)	99.7 (99.7,76.8)	92.1 (79.6,50.6)	99.7 (7.9,99.4)
	**joe biden**	86.3 (0.9,86.3)	98.8 (82,94.2)	99.7 (99.7,80.8)	89 (77.4,52.1)	99.7 (7.9,99.7)
	**us elections**	96.3 (1.2,96.3)	100 (100,99.4)	100 (100,79.3)	93.6 (79.6,54)	100 (7.9,100)

Table S2. Coverage of collection B. The table shows the coverage per region (first column), query term (second column) and search engine (first row). The main value of each cell shows the percentage of rounds for which search results were collected; the first value in the parenthesis shows the percentage of rounds for which search results were collected for the Firefox agent and the second value does for the Chrome agent.

Although we targeted the top 50 results, this was not always achieved, see Table S3 for averages of the coverage per condition. For most of the cases, we collected 45 results on average. Occasionally (.35%) there are gaps between the top-X results due to a missing page of the pagination sequence attributed to network failures: 1.09% for Baidu, .77% for Google and .03% for Yahoo. There were very particular cases in which there were more systematics issues with the coverage. For DuckDuckGo, the averages ranged between 21 and 30 results due to a potential error ("bug") in the search engine where loading results more than twice is not

always possible for news results[1] (the bug affected all our queries except “coronavirus”). For “nagorno-karabakh conflict” and “poland abortion” for Bing in Frankfurt, we only collected on average 6 and 9 results respectively, but this is attributed to the lack of items available in Bing’s engine. For a similar reason but likely confounded with the language of the queries, the number of results was low (between 6 and 21) for all queries but

		**Baidu**				**Bing**				**DuckDuckGo**				**Google**				**Yahoo!**			
		**Frankfurt**		**Oregon**		**Frankfurt**		**Oregon**		**Frankfurt**		**Oregon**		**Frankfurt**		**Oregon**		**Frankfurt**		**Oregon**	
		**M**	**Mdn**	**M**	**Mdn**	**M**	**Mdn**	**M**	**Mdn**	**M**	**Mdn**	**M**	**Mdn**	**M**	**Mdn**	**M**	**Mdn**	**M**	**Mdn**	**M**	**Mdn**
**elections**	**donald trump**	45.8	45	44.7	46	46	50	50	50	26	26	22.9	23	46.8	49	48.7	50	50	50	50	50
	**joe biden**	48.1	48	45.9	48	45.9	50	50	50	31.6	29	27.1	26	47.4	49	48.5	50	50	50	50	50
	**us elections**	18.5	19	15.3	18	46.3	50	50	50	25.2	24	22.4	21	49.2	50	49.3	50	50	50	50	50
**topical**	**coronavirus**	37.9	38	38.6	38	43.3	50	46.7	50	45.1	50	44.9	50	50	50	48.8	50	50	50	50	50
	**nagorno-karabakh conflict**	6	6	5.7	6	6	6	41.7	50	28.6	29	28.8	28	50	50	49.9	50	50	50	50	50
	**poland abortion**	6	6	6	6	9	9	48.9	50	21	22	20.6	22	50	50	49.8	50	49.9	50	50	50
**stable**	**first world war**	10.5	10	10.8	10	47	50	50	50	31.9	29	29.6	28	50	50	49.9	50	50	50	50	50
	**holocaust**	8.8	9	8.8	9	47.7	50	50	50	28.5	29	28.3	28	50	50	49.9	50	50	50	50	50
	**virtual reality**	20.6	21	20.7	21	46.9	50	50	50	28.4	28	28.4	28	50	50	49.8	50	49.9	50	50	50

Table S3. Means and medians of the top-x results collected. The table shows the mean and median per query category (first column), query term (second column), search engine (first row), region (second row).

[1] To load more results in DuckDuckGo, the agent “clicks” a button that appears at the bottom of the results. It is often the case that the button does not re-appear after clicking it once, even for current events for which there are certainly more news results. This behavior is not consistent as for most queries we managed to collect 50 results in some of the rounds regardless of the browser or region.

Appendix S4. Linear mixed model, ANOVA and contrasts tables for the novelty based on the categories of the queries

```
Linear mixed model fit by REML. t-tests use Satterthwaite's method ['lmerModLmerTest']
Formula: weighted_novelty ~ query_type * engine * place + (1 | query) + (1 | round) + (1 | agent)

REML criterion at convergence: 2343601

Scaled residuals:
       Min      1Q  Median       3Q      Max
-0.9741 -0.3021 -0.1129 -0.0226 10.8891

Random effects:
 Groups   Name          Variance  Std.Dev.
 round (Intercept) 1.675e-03 0.040925
 agent (Intercept) 4.949e-05 0.007035
 query (Intercept) 1.541e-03 0.039252
 Residual              1.739e-01 0.416991
Number of obs: 2151349, groups:  round, 409; agent, 223; query, 9

Fixed effects: (see ANOVA table and Contrasts instead)
```

```
ANOVA Table [anova(the_model)]:
                        Sum Sq Mean Sq NumDF   DenDF  F value Pr(>F) sig
query_type               2.970   1.485     2   6.025    8.540  0.017   *
engine                 836.228 209.057     4 211.114 1202.295 <0.001 ***
place                   21.572  21.572     1 224.674  124.063 <0.001 ***
query_type:engine      398.933  49.867     8 208.750  286.785 <0.001 ***
query_type:place        13.312   6.656     2 222.458   38.280 <0.001 ***
engine:place            41.968  10.492     4 210.306   60.341 <0.001 ***
query_type:engine:place 12.805   1.601     8 207.966    9.205 <0.001 ***
```

```
H1a & H1b - Contrasts Table [emmeans(the_model,  ~ permeator|(period:engine), lmer.df = "asymptotic")]:
    engine     place query_type_pairwise estimate     SE  df asymp.LCL asymp.UCL z.ratio p.value sig

H1a - US Elections category vs Topical (fluid) category:
      Baidu    Oregon   elections - fluid   0.0356 0.0324 Inf   -0.0663    0.1375  1.0981  >0.999
      Baidu Frankfurt   elections - fluid   0.0314 0.0325 Inf   -0.0709    0.1336  0.9643  >0.999
       Bing    Oregon   elections - fluid   0.1513 0.0323 Inf    0.0497    0.2529  4.6814  <0.001 ***
       Bing Frankfurt   elections - fluid   0.1188 0.0324 Inf    0.0168    0.2209  3.6625   0.007  **
 DuckDuckGo    Oregon   elections - fluid   0.1203 0.0324 Inf    0.0186    0.2221  3.7173   0.006  **
 DuckDuckGo Frankfurt   elections - fluid   0.0841 0.0324 Inf   -0.0178    0.1860  2.5949   0.284
     Google    Oregon   elections - fluid   0.1511 0.0323 Inf    0.0495    0.2527  4.6767  <0.001 ***
     Google Frankfurt   elections - fluid   0.1231 0.0323 Inf    0.0214    0.2248  3.8065   0.004  **
     Yahoo!    Oregon   elections - fluid   0.0577 0.0323 Inf   -0.0438    0.1592  1.7865  >0.999
     Yahoo! Frankfurt   elections - fluid   0.0605 0.0325 Inf   -0.0416    0.1626  1.8627  >0.999

H1a - US Elections category vs Stable category:
      Baidu    Oregon  elections - stable   0.0092 0.0325 Inf   -0.0929    0.1113  0.2833  >0.999
      Baidu Frankfurt  elections - stable   0.0043 0.0325 Inf   -0.0979    0.1064  0.1320  >0.999
       Bing    Oregon  elections - stable   0.2227 0.0323 Inf    0.1212    0.3243  6.8947  <0.001 ***
       Bing Frankfurt  elections - stable   0.1630 0.0324 Inf    0.0612    0.2647  5.0347  <0.001 ***
 DuckDuckGo    Oregon  elections - stable   0.1972 0.0324 Inf    0.0955    0.2989  6.0944  <0.001 ***
 DuckDuckGo Frankfurt  elections - stable   0.1716 0.0324 Inf    0.0697    0.2735  5.2960  <0.001 ***
     Google    Oregon  elections - stable   0.1818 0.0323 Inf    0.0802    0.2833  5.6265  <0.001 ***
     Google Frankfurt  elections - stable   0.1512 0.0323 Inf    0.0496    0.2528  4.6779  <0.001 ***
     Yahoo!    Oregon  elections - stable   0.0891 0.0323 Inf   -0.0124    0.1906  2.7592   0.174
     Yahoo! Frankfurt  elections - stable   0.0920 0.0325 Inf   -0.0101    0.1942  2.8329   0.138

H1b - Topical (fluid) category vs Stable category:
      Baidu    Oregon      fluid - stable  -0.0264 0.0326 Inf   -0.1288    0.0760 -0.8109  >0.999
      Baidu Frankfurt      fluid - stable  -0.0271 0.0326 Inf   -0.1296    0.0755 -0.8302  >0.999
       Bing    Oregon      fluid - stable   0.0714 0.0323 Inf   -0.0302    0.1730  2.2084   0.817
       Bing Frankfurt      fluid - stable   0.0441 0.0324 Inf   -0.0579    0.1461  1.3597  >0.999
 DuckDuckGo    Oregon      fluid - stable   0.0769 0.0324 Inf   -0.0248    0.1786  2.3763   0.525
 DuckDuckGo Frankfurt      fluid - stable   0.0875 0.0324 Inf   -0.0142    0.1893  2.7046   0.205
     Google    Oregon      fluid - stable   0.0306 0.0323 Inf   -0.0709    0.1322  0.9482  >0.999
     Google Frankfurt      fluid - stable   0.0281 0.0323 Inf   -0.0735    0.1297  0.8690  >0.999
     Yahoo!    Oregon      fluid - stable   0.0314 0.0323 Inf   -0.0701    0.1330  0.9726  >0.999
     Yahoo! Frankfurt      fluid - stable   0.0315 0.0325 Inf   -0.0708    0.1338  0.9683  >0.999

Degrees-of-freedom method: asymptotic
Confidence level used: 0.95
Conf-level adjustment: bonferroni method for 30 estimates
P value adjustment: bonferroni method for 30 tests
```

Table S4.Linear mixed model, ANOVA and contrasts table for the novelty based on the categories of the queries. The top row of the table presents the linear mixed model, the general resulting statistics and random effect statistics. The fixed effects combinations are omitted and instead the ANOVA Table (middle row) is presented. The third row presents the within period-engine contrasts to tests H1a and H1b. Contrasts are highlighted green if they support the hypothesis, yellow if they discard the hypothesis, and red if the effect is in the opposite direction of the predicted one.

Appendix S5. Linear mixed model, ANOVA and contrasts table for the novelty based on the categories (including the new coronavirus category)

```
Linear mixed model fit by REML. t-tests use Satterthwaite's method ['lmerModLmerTest']
Formula: weighted_novelty ~ query_type * engine * place + (1 | query) +      (1 | round) + (1 | agent)
   Data: df

REML criterion at convergence: 2340973

Scaled residuals:
    Min      1Q  Median      3Q     Max
-0.9747 -0.2972 -0.1004 -0.0313 10.8966

Random effects:
 Groups   Name        Variance  Std.Dev.
 round    (Intercept) 1.669e-03 0.040859
 agent    (Intercept) 4.611e-05 0.006791
 query    (Intercept) 2.370e-04 0.015395
 Residual             1.737e-01 0.416731
Number of obs: 2151349, groups:  round, 409; agent, 223; query, 9

Fixed effects: (see ANOVA table and Contrasts instead)
```

```
ANOVA Table [anova(the_model)]:
                        Sum Sq Mean Sq NumDF   DenDF F value Pr(>F) sig
query_type               24.003   8.001     3   5.093  46.072 <0.001 ***
engine                  689.492 172.373     4 246.709 992.561 <0.001 ***
place                    21.137  21.137     1 273.435 121.713 <0.001 ***
query_type:engine       789.121  65.760    12 423.968 378.661 <0.001 ***
query_type:place         14.768   4.923     3 450.633  28.347 <0.001 ***
engine:place             52.353  13.088     4 246.189  75.364 <0.001 ***
query_type:engine:place  34.162   2.847    12 423.766  16.393 <0.001 ***
```

```
H1a & H1b - Contrasts Table [emmeans(the_model,  ~ permeator|(period:engine), lmer.df = "asymptotic"))]:
    engine     place     query_type_pairwise estimate     SE  df asymp.LCL asymp.UCL z.ratio p.value sig

H1a - US Elections category vs Coronavirus category:
      Baidu    Oregon coronavirus - elections   0.0146 0.0185 Inf   -0.0472    0.0764  0.7917  >0.999
      Baidu Frankfurt coronavirus - elections   0.0169 0.0187 Inf   -0.0456    0.0794  0.9026  >0.999
       Bing    Oregon coronavirus - elections  -0.0137 0.0184 Inf   -0.0752    0.0477 -0.7470  >0.999
       Bing Frankfurt coronavirus - elections  -0.0374 0.0185 Inf   -0.0994    0.0245 -2.0189  >0.999
 DuckDuckGo    Oregon coronavirus - elections   0.0005 0.0185 Inf   -0.0612    0.0622  0.0250  >0.999
 DuckDuckGo Frankfurt coronavirus - elections   0.0323 0.0185 Inf   -0.0295    0.0941  1.7472  >0.999
     Google    Oregon coronavirus - elections  -0.1213 0.0184 Inf   -0.1827   -0.0599 -6.5964  <0.001 ***
     Google Frankfurt coronavirus - elections  -0.0840 0.0184 Inf   -0.1454   -0.0225 -4.5651  <0.001 ***
     Yahoo!    Oregon coronavirus - elections  -0.0058 0.0183 Inf   -0.0669    0.0554 -0.3145  >0.999
     Yahoo! Frankfurt coronavirus - elections  -0.0083 0.0187 Inf   -0.0708    0.0542 -0.4445  >0.999

H1a - US Elections category vs Topical (fluid) category:
     engine      place query_type_pairwise estimate     SE  df asymp.LCL asymp.UCL z.ratio p.value sig
      Baidu    Oregon   elections - fluid   0.0101 0.0156 Inf   -0.0420    0.0623  0.6486  >0.999
      Baidu Frankfurt   elections - fluid   0.0063 0.0160 Inf   -0.0470    0.0596  0.3919  >0.999
       Bing    Oregon   elections - fluid   0.2192 0.0147 Inf    0.1701    0.2682 14.9301  <0.001 ***
       Bing Frankfurt   elections - fluid   0.1740 0.0155 Inf    0.1223    0.2258 11.2348  <0.001 ***
 DuckDuckGo    Oregon   elections - fluid   0.1896 0.0148 Inf    0.1400    0.2391 12.7902  <0.001 ***
 DuckDuckGo Frankfurt   elections - fluid   0.1604 0.0149 Inf    0.1106    0.2103 10.7464  <0.001 ***
     Google    Oregon   elections - fluid   0.1713 0.0146 Inf    0.1224    0.2202 11.6964  <0.001 ***
     Google Frankfurt   elections - fluid   0.1429 0.0147 Inf    0.0938    0.1921  9.7156  <0.001 ***
     Yahoo!    Oregon   elections - fluid   0.0837 0.0146 Inf    0.0349    0.1325  5.7288  <0.001 ***
     Yahoo! Frankfurt   elections - fluid   0.0868 0.0150 Inf    0.0365    0.1370  5.7726  <0.001 ***

H1a - US Elections category vs Stable category:
      Baidu    Oregon  elections - stable   0.0092 0.0136 Inf   -0.0361    0.0545  0.6767  >0.999
      Baidu Frankfurt  elections - stable   0.0043 0.0136 Inf   -0.0411    0.0498  0.3190  >0.999
       Bing    Oregon  elections - stable   0.2226 0.0132 Inf    0.1786    0.2666 16.9019  <0.001 ***
       Bing Frankfurt  elections - stable   0.1629 0.0133 Inf    0.1184    0.2074 12.2333  <0.001 ***
 DuckDuckGo    Oregon  elections - stable   0.1971 0.0133 Inf    0.1526    0.2416 14.8162  <0.001 ***
 DuckDuckGo Frankfurt  elections - stable   0.1717 0.0134 Inf    0.1268    0.2165 12.7987  <0.001 ***
     Google    Oregon  elections - stable   0.1817 0.0132 Inf    0.1377    0.2258 13.7978  <0.001 ***
     Google Frankfurt  elections - stable   0.1512 0.0132 Inf    0.1071    0.1953 11.4460  <0.001 ***
     Yahoo!    Oregon  elections - stable   0.0891 0.0132 Inf    0.0452    0.1331  6.7766  <0.001 ***
     Yahoo! Frankfurt  elections - stable   0.0921 0.0136 Inf    0.0467    0.1375  6.7764  <0.001 ***

H1b - Topical (fluid) category vs Stable category:
      Baidu    Oregon      fluid - stable  -0.0010 0.0159 Inf   -0.0541    0.0522 -0.0598  >0.999
      Baidu Frankfurt      fluid - stable  -0.0019 0.0161 Inf   -0.0558    0.0520 -0.1183  >0.999
       Bing    Oregon      fluid - stable   0.0034 0.0147 Inf   -0.0457    0.0525  0.2334  >0.999
       Bing Frankfurt      fluid - stable  -0.0111 0.0155 Inf   -0.0628    0.0406 -0.7164  >0.999
 DuckDuckGo    Oregon      fluid - stable   0.0075 0.0148 Inf   -0.0419    0.0570  0.5096  >0.999
 DuckDuckGo Frankfurt      fluid - stable   0.0112 0.0148 Inf   -0.0384    0.0608  0.7564  >0.999
     Google    Oregon      fluid - stable   0.0104 0.0146 Inf   -0.0385    0.0594  0.7133  >0.999
     Google Frankfurt      fluid - stable   0.0083 0.0147 Inf   -0.0407    0.0573  0.5652  >0.999
```

```
     Yahoo!    Oregon      fluid - stable   0.0054 0.0146 Inf   -0.0434    0.0543  0.3720  >0.999
     Yahoo! Frankfurt      fluid - stable   0.0053 0.0152 Inf   -0.0454    0.0560  0.3494  >0.999

Degrees-of-freedom method: asymptotic
Confidence level used: 0.95
Conf-level adjustment: bonferroni method for 60 estimates
P value adjustment: bonferroni method for 60 tests
Showing 40 out of 60 tests: coronavirus - (fluid|stable) contrasts are not shown
```

TableS5. Linear mixed model, ANOVA and contrasts table for the novelty based on the categories (including the new coronavirus category). The top row of the table presents the linear mixed model, the general resulting statistics and random effect statistics. The fixed effects combinations are omitted and instead the ANOVA Table (middle row) is presented. The third row presents the category contrasts within period-engine to test H1a and H2b. Contrasts are highlighted green if they support the hypothesis, yellow if they discard the hypothesis, and red if the effect is in the opposite direction of the predicted one.

Appendix S6. Linear mixed model, ANOVA and contrasts tables for the novelty of the US-related queries

```
Linear mixed model fit by REML. t-tests use Satterthwaite's method ['lmerModLmerTest']
Formula: weighted_novelty ~ query * engine * place * period + (1 | agent) +      (1 | round) + (1 | agent:round)

REML criterion at convergence: 1484865

Scaled residuals:
    Min      1Q  Median      3Q     Max
-0.9721 -0.3319 -0.2133 -0.0993  8.3275

Random effects:
 Groups      Name        Variance  Std.Dev.
 agent:round (Intercept) 0.0002494 0.01579
 round       (Intercept) 0.0012546 0.03542
 agent       (Intercept) 0.0000297 0.00545
 Residual                0.2941596 0.54236
Number of obs: 918415, groups:  agent:round, 7997; round, 408; agent, 59

Fixed effects: (see ANOVA table and Contrasts instead)
```

ANOVA Table [anova(the_model)]:

	Sum Sq	Mean Sq	NumDF	DenDF	F value	Pr(>F)	sig
query	203.077	101.539	2	768776.685	345.182	<0.001	***
engine	444.493	148.164	3	30.620	503.687	<0.001	***
place	41.246	41.246	1	31.527	140.216	<0.001	***
period	68.976	22.992	3	413.632	78.161	<0.001	***
query:engine	351.989	58.665	6	775622.148	199.432	<0.001	***
query:place	245.468	122.734	2	768329.415	417.236	<0.001	***
engine:place	27.917	9.306	3	30.529	31.634	<0.001	***
query:period	171.747	28.625	6	775006.007	97.309	<0.001	***
engine:period	38.968	4.330	9	1238.391	14.719	<0.001	***
place:period	9.221	3.074	3	1241.880	10.449	<0.001	***
query:engine:place	651.178	108.530	6	775076.030	368.948	<0.001	***
query:engine:period	232.435	12.913	18	790136.288	43.898	<0.001	***
query:place:period	59.696	9.949	6	774340.570	33.823	<0.001	***
engine:place:period	37.446	4.161	9	1237.092	14.144	<0.001	***
query:engine:place:period	92.405	5.134	18	789420.858	17.452	<0.001	***

H2a - Contrasts Table [emmeans(the_model, ~ place|(engine:query:period), lmer.df = "asymptotic")]:

query	engine	period	place_pairwise	estimate	SE	df	asymp.LCL	asymp.UCL	z.ratio	p.value	sig
**"donald trump" query:**											
donald trump	Bing	I	Frankfurt - Oregon	-0.1049	0.0074	Inf	-0.1293	-0.0806	-14.1330	<0.001	***
donald trump	DuckDuckGo	I	Frankfurt - Oregon	-0.0307	0.0096	Inf	-0.0622	0.0007	-3.2078	0.064	
donald trump	Google	I	Frankfurt - Oregon	-0.0396	0.0075	Inf	-0.0640	-0.0151	-5.3083	<0.001	***
donald trump	Yahoo!	I	Frankfurt - Oregon	0.0031	0.0076	Inf	-0.0219	0.0281	0.4110	>0.999	
donald trump	Bing	II	Frankfurt - Oregon	-0.0187	0.0081	Inf	-0.0452	0.0078	-2.3189	0.979	
donald trump	DuckDuckGo	II	Frankfurt - Oregon	-0.0545	0.0127	Inf	-0.0963	-0.0127	-4.2746	<0.001	***
donald trump	Google	II	Frankfurt - Oregon	0.0374	0.0090	Inf	0.0080	0.0668	4.1743	0.001	**
donald trump	Yahoo!	II	Frankfurt - Oregon	0.0008	0.0085	Inf	-0.0270	0.0287	0.0989	>0.999	
donald trump	Bing	III	Frankfurt - Oregon	-0.1002	0.0072	Inf	-0.1239	-0.0765	-13.8373	<0.001	***
donald trump	DuckDuckGo	III	Frankfurt - Oregon	-0.0288	0.0127	Inf	-0.0704	0.0127	-2.2746	>0.999	
donald trump	Google	III	Frankfurt - Oregon	-0.0081	0.0099	Inf	-0.0406	0.0243	-0.8217	>0.999	
donald trump	Yahoo!	III	Frankfurt - Oregon	-0.0001	0.0085	Inf	-0.0280	0.0278	-0.0070	>0.999	
donald trump	Bing	IV	Frankfurt - Oregon	-0.0561	0.0102	Inf	-0.0896	-0.0227	-5.4997	<0.001	***
donald trump	DuckDuckGo	IV	Frankfurt - Oregon	-0.0239	0.0158	Inf	-0.0757	0.0279	-1.5117	>0.999	
donald trump	Google	IV	Frankfurt - Oregon	-0.0542	0.0130	Inf	-0.0969	-0.0115	-4.1624	0.002	**
donald trump	Yahoo!	IV	Frankfurt - Oregon	-0.0010	0.0100	Inf	-0.0339	0.0318	-0.1035	>0.999	
**"joe biden" query:**											
joe biden	Bing	I	Frankfurt - Oregon	0.1121	0.0079	Inf	0.0863	0.1380	14.2148	<0.001	***
joe biden	DuckDuckGo	I	Frankfurt - Oregon	-0.0009	0.0095	Inf	-0.0321	0.0302	-0.0966	>0.999	
joe biden	Google	I	Frankfurt - Oregon	-0.0620	0.0073	Inf	-0.0860	-0.0379	-8.4605	<0.001	***
joe biden	Yahoo!	I	Frankfurt - Oregon	0.0055	0.0076	Inf	-0.0195	0.0305	0.7165	>0.999	
joe biden	Bing	II	Frankfurt - Oregon	0.1471	0.0087	Inf	0.1187	0.1755	16.9932	<0.001	***
joe biden	DuckDuckGo	II	Frankfurt - Oregon	-0.0076	0.0124	Inf	-0.0482	0.0331	-0.6104	>0.999	
joe biden	Google	II	Frankfurt - Oregon	0.0084	0.0086	Inf	-0.0197	0.0366	0.9814	>0.999	
joe biden	Yahoo!	II	Frankfurt - Oregon	0.0123	0.0084	Inf	-0.0153	0.0398	1.4602	>0.999	
joe biden	Bing	III	Frankfurt - Oregon	0.0365	0.0076	Inf	0.0116	0.0614	4.8025	<0.001	***
joe biden	DuckDuckGo	III	Frankfurt - Oregon	-0.0129	0.0112	Inf	-0.0496	0.0238	-1.1533	>0.999	
joe biden	Google	III	Frankfurt - Oregon	-0.0231	0.0097	Inf	-0.0549	0.0087	-2.3833	0.824	
joe biden	Yahoo!	III	Frankfurt - Oregon	0.0042	0.0085	Inf	-0.0238	0.0322	0.4919	>0.999	
joe biden	Bing	IV	Frankfurt - Oregon	0.1408	0.0108	Inf	0.1054	0.1761	13.0528	<0.001	***
joe biden	DuckDuckGo	IV	Frankfurt - Oregon	0.0142	0.0147	Inf	-0.0341	0.0624	0.9618	>0.999	
joe biden	Google	IV	Frankfurt - Oregon	-0.0435	0.0130	Inf	-0.0862	-0.0008	-3.3404	0.040	*
joe biden	Yahoo!	IV	Frankfurt - Oregon	0.0032	0.0100	Inf	-0.0296	0.0361	0.3215	>0.999	
**"US elections" query:**											
us elections	Bing	I	Frankfurt - Oregon	-0.2440	0.0075	Inf	-0.2686	-0.2193	-32.4348	<0.001	***

```
 us elections DuckDuckGo       I Frankfurt - Oregon  -0.0698 0.0110 Inf   -0.1057    -0.0338   -6.3641  <0.001 ***
 us elections     Google       I Frankfurt - Oregon  -0.0400 0.0075 Inf   -0.0646    -0.0154   -5.3271  <0.001 ***
 us elections     Yahoo!       I Frankfurt - Oregon  -0.0013 0.0076 Inf   -0.0263     0.0237   -0.1707  >0.999
 us elections       Bing      II Frankfurt - Oregon  -0.3082 0.0082 Inf   -0.3352    -0.2811  -37.3689 <0.001 ***
 us elections DuckDuckGo      II Frankfurt - Oregon  -0.0717 0.0130 Inf   -0.1144    -0.0290   -5.5029  <0.001 ***
 us elections     Google      II Frankfurt - Oregon  -0.0599 0.0090 Inf   -0.0895    -0.0304   -6.6526  <0.001 ***
 us elections     Yahoo!      II Frankfurt - Oregon  -0.0042 0.0083 Inf   -0.0315     0.0231   -0.5037  >0.999
 us elections       Bing     III Frankfurt - Oregon  -0.1519 0.0072 Inf   -0.1757    -0.1282  -20.9859 <0.001 ***
 us elections DuckDuckGo     III Frankfurt - Oregon  -0.0036 0.0133 Inf   -0.0471     0.0400   -0.2693  >0.999
 us elections     Google     III Frankfurt - Oregon  -0.0192 0.0099 Inf   -0.0515     0.0132   -1.9444  >0.999
 us elections     Yahoo!     III Frankfurt - Oregon  -0.0029 0.0085 Inf   -0.0308     0.0250   -0.3414  >0.999
 us elections       Bing      IV Frankfurt - Oregon  -0.1245 0.0101 Inf   -0.1577    -0.0912  -12.2769 <0.001 ***
 us elections DuckDuckGo      IV Frankfurt - Oregon   0.0263 0.0140 Inf   -0.0197     0.0723    1.8758  >0.999
 us elections     Google      IV Frankfurt - Oregon  -0.0646 0.0128 Inf   -0.1065    -0.0227   -5.0555  <0.001 ***
 us elections     Yahoo!      IV Frankfurt - Oregon  -0.0011 0.0100 Inf   -0.0339     0.0316   -0.1148  >0.999


Results are averaged over some or all of the levels of: period
Degrees-of-freedom method: asymptotic
Confidence level used: 0.95
Conf-level adjustment: bonferroni method for 12 estimates
P value adjustment: bonferroni method for 12 tests
```

H2b & H2c - Contrasts Table: [emmeans(the_model, ~ engine|(query:place), lmer.df = "asymptotic"),at = list(place = c("Oregon"))]:

```
    place        query     engine_pairwise estimate     SE  df asymp.LCL asymp.UCL  z.ratio p.value sig
  Contrasts for Oregon (in consecutive order of the means showed below):
    Oregon donald trump   Bing - DuckDuckGo   0.0254 0.0057 Inf    0.0071    0.0437   4.4463  <0.001 ***
    Oregon donald trump DuckDuckGo - Google   0.0746 0.0063 Inf    0.0546    0.0946  11.9278  <0.001 ***
    Oregon donald trump     Google - Yahoo!   0.0716 0.0055 Inf    0.0539    0.0892  12.9412  <0.001 ***
    Oregon    joe biden DuckDuckGo - Google  -0.0307 0.0060 Inf   -0.0500   -0.0113  -5.0732  <0.001 ***
    Oregon    joe biden DuckDuckGo - Yahoo!   0.0520 0.0058 Inf    0.0334    0.0707   8.9071  <0.001 ***
    Oregon    joe biden       Bing - Yahoo!  -0.0016 0.0051 Inf   -0.0180    0.0148  -0.3073  >0.999
    Oregon us elections   Bing - DuckDuckGo   0.0230 0.0058 Inf    0.0046    0.0415   3.9983   0.002  **
    Oregon us elections DuckDuckGo - Google   0.0870 0.0062 Inf    0.0672    0.1068  14.0373  <0.001 ***
    Oregon us elections     Google - Yahoo!   0.0954 0.0055 Inf    0.0779    0.1128  17.4771  <0.001 ***

  Contrasts for Frankfurt (in consecutive order of the means showed below):
 Frankfurt donald trump   Bing - DuckDuckGo  -0.0101 0.0067 Inf   -0.0315    0.0112  -1.5149  >0.999
 Frankfurt donald trump       Bing - Google   0.0461 0.0057 Inf    0.0280    0.0642   8.1509  <0.001 ***
 Frankfurt donald trump     Google - Yahoo!   0.0547 0.0056 Inf    0.0370    0.0725   9.8526  <0.001 ***
 Frankfurt    joe biden       Bing - Google   0.0549 0.0056 Inf    0.0370    0.0727   9.8260  <0.001 ***
 Frankfurt    joe biden DuckDuckGo - Google  -0.0024 0.0065 Inf   -0.0233    0.0184  -0.3748  >0.999
 Frankfurt    joe biden DuckDuckGo - Yahoo!   0.0439 0.0064 Inf    0.0236    0.0643   6.9118  <0.001 ***
 Frankfurt us elections DuckDuckGo - Google   0.1033 0.0068 Inf    0.0817    0.1249  15.2726  <0.001 ***
 Frankfurt us elections       Bing - Google  -0.0512 0.0056 Inf   -0.0692   -0.0332  -9.0869  <0.001 ***
 Frankfurt us elections       Bing - Yahoo!   0.0006 0.0054 Inf   -0.0165    0.0178   0.1211  >0.999

Results are averaged over all of the levels of: period
Degrees-of-freedom method: asymptotic
Confidence level used: 0.95
Conf-level adjustment: bonferroni method for 18 estimates
P value adjustment: bonferroni method for 18 tests
Showing 18 out of 36 tests ordered by their means (showed below)


 Sorted novelty means for Oregon
query = donald trump, place = Oregon:
 engine      emmean      SE  df asymp.LCL asymp.UCL
 Bing        0.2584 0.00382 Inf    0.2509    0.2659
 DuckDuckGo  0.2330 0.00517 Inf    0.2229    0.2431
 Google      0.1584 0.00459 Inf    0.1494    0.1674
 Yahoo!      0.0869 0.00428 Inf    0.0785    0.0953

query = joe biden, place = Oregon:
 engine      emmean      SE  df asymp.LCL asymp.UCL
 Google      0.1885 0.00456 Inf    0.1796    0.1974
 DuckDuckGo  0.1578 0.00495 Inf    0.1481    0.1675
 Yahoo!      0.1058 0.00428 Inf    0.0974    0.1142
 Bing        0.1042 0.00410 Inf    0.0962    0.1122

query = us elections, place = Oregon:
 engine      emmean      SE  df asymp.LCL asymp.UCL
 Bing        0.2470 0.00388 Inf    0.2394    0.2546
 DuckDuckGo  0.2240 0.00519 Inf    0.2138    0.2341
 Google      0.1370 0.00451 Inf    0.1281    0.1458
 Yahoo!      0.0416 0.00427 Inf    0.0332    0.0500

 Sorted novelty means for Frankfurt
query = donald trump, place = Frankfurt:
 engine      emmean      SE  df asymp.LCL asymp.UCL
 DuckDuckGo  0.1985 0.00580 Inf    0.1871    0.2099
```

engine	emmean	SE	df	asymp.LCL	asymp.UCL
Bing	0.1884	0.00443	Inf	0.1797	0.1971
Google	0.1423	0.00461	Inf	0.1333	0.1513
Yahoo!	0.0876	0.00428	Inf	0.0792	0.0960

query = joe biden, place = Frankfurt:

engine	emmean	SE	df	asymp.LCL	asymp.UCL
Bing	0.2133	0.00442	Inf	0.2047	0.2220
Google	0.1585	0.00453	Inf	0.1496	0.1673
DuckDuckGo	0.1560	0.00556	Inf	0.1451	0.1669
Yahoo!	0.1121	0.00427	Inf	0.1037	0.1204

query = us elections, place = Frankfurt:

engine	emmean	SE	df	asymp.LCL	asymp.UCL
DuckDuckGo	0.1943	0.00577	Inf	0.1830	0.2056
Google	0.0910	0.00461	Inf	0.0820	0.1001
Bing	0.0399	0.00439	Inf	0.0313	0.0485
Yahoo!	0.0392	0.00426	Inf	0.0309	0.0476

H2d - Contrasts Table [emmeans(the_model, ~ period|(query:engine), lmer.df = "asymptotic", at = list(place = c("Oregon")))

Oregon - "donald trump" query:

place	query	engine	period_pairwise	estimate	SE	df	asymp.LCL	asymp.UCL	z.ratio	p.value	sig
Oregon	donald trump	Bing	I - II	0.0364	0.0095	Inf	0.0024	0.0704	3.8348	0.018	*
Oregon	donald trump	Bing	I - III	0.0903	0.0084	Inf	0.0602	0.1204	10.7286	<0.001	***
Oregon	donald trump	Bing	I - IV	0.1358	0.0091	Inf	0.1032	0.1683	14.9248	<0.001	***
Oregon	donald trump	Bing	II - III	0.0539	0.0087	Inf	0.0229	0.0848	6.2210	<0.001	***
Oregon	donald trump	Bing	II - IV	0.0994	0.0093	Inf	0.0660	0.1327	10.6581	<0.001	***
Oregon	donald trump	Bing	III - IV	0.0455	0.0076	Inf	0.0182	0.0728	5.9663	<0.001	***
Oregon	donald trump	DuckDuckGo	I - II	0.0087	0.0124	Inf	-0.0358	0.0532	0.7011	>0.999	
Oregon	donald trump	DuckDuckGo	I - III	0.0341	0.0120	Inf	-0.0087	0.0768	2.8503	0.629	
Oregon	donald trump	DuckDuckGo	I - IV	0.0524	0.0133	Inf	0.0047	0.1001	3.9301	0.012	*
Oregon	donald trump	DuckDuckGo	II - III	0.0254	0.0129	Inf	-0.0206	0.0713	1.9732	>0.999	
Oregon	donald trump	DuckDuckGo	II - IV	0.0437	0.0141	Inf	-0.0069	0.0943	3.0891	0.289	
Oregon	donald trump	DuckDuckGo	III - IV	0.0183	0.0133	Inf	-0.0292	0.0659	1.3812	>0.999	
Oregon	donald trump	Google	I - II	0.0002	0.0102	Inf	-0.0362	0.0366	0.0184	>0.999	
Oregon	donald trump	Google	I - III	0.0464	0.0099	Inf	0.0110	0.0818	4.6922	<0.001	***
Oregon	donald trump	Google	I - IV	0.0373	0.0121	Inf	-0.0059	0.0806	3.0877	0.290	
Oregon	donald trump	Google	II - III	0.0462	0.0104	Inf	0.0090	0.0835	4.4445	0.001	**
Oregon	donald trump	Google	II - IV	0.0371	0.0125	Inf	-0.0077	0.0819	2.9657	0.435	
Oregon	donald trump	Google	III - IV	-0.0091	0.0118	Inf	-0.0515	0.0332	-0.7700	>0.999	
Oregon	donald trump	Yahoo!	I - II	0.0069	0.0095	Inf	-0.0271	0.0409	0.7250	>0.999	
Oregon	donald trump	Yahoo!	I - III	0.0609	0.0095	Inf	0.0269	0.0949	6.4129	<0.001	***
Oregon	donald trump	Yahoo!	I - IV	0.0650	0.0106	Inf	0.0269	0.1030	6.1071	<0.001	***
Oregon	donald trump	Yahoo!	II - III	0.0540	0.0097	Inf	0.0192	0.0889	5.5437	<0.001	***
Oregon	donald trump	Yahoo!	II - IV	0.0581	0.0109	Inf	0.0192	0.0969	5.3481	<0.001	***
Oregon	donald trump	Yahoo!	III - IV	0.0041	0.0099	Inf	-0.0314	0.0395	0.4093	>0.999	

(11 in favour, 0 against, 24 in total)

Oregon - "joe biden" query:

place	query	engine	period_pairwise	estimate	SE	df	asymp.LCL	asymp.UCL	z.ratio	p.value	sig
Oregon	joe biden	Bing	I - II	0.0172	0.0104	Inf	-0.0200	0.0544	1.6581	>0.999	
Oregon	joe biden	Bing	I - III	0.0137	0.0092	Inf	-0.0192	0.0466	1.4892	>0.999	
Oregon	joe biden	Bing	I - IV	-0.0311	0.0102	Inf	-0.0677	0.0054	-3.0455	0.335	
Oregon	joe biden	Bing	II - III	-0.0036	0.0095	Inf	-0.0376	0.0304	-0.3737	>0.999	
Oregon	joe biden	Bing	II - IV	-0.0484	0.0105	Inf	-0.0859	-0.0108	-4.6042	<0.001	***
Oregon	joe biden	Bing	III - IV	-0.0448	0.0088	Inf	-0.0762	-0.0135	-5.1145	<0.001	***
Oregon	joe biden	DuckDuckGo	I - II	0.0358	0.0122	Inf	-0.0078	0.0795	2.9348	0.481	
Oregon	joe biden	DuckDuckGo	I - III	0.0981	0.0112	Inf	0.0581	0.1381	8.7787	<0.001	***
Oregon	joe biden	DuckDuckGo	I - IV	0.0087	0.0128	Inf	-0.0370	0.0544	0.6792	>0.999	
Oregon	joe biden	DuckDuckGo	II - III	0.0623	0.0120	Inf	0.0193	0.1052	5.1901	<0.001	***
Oregon	joe biden	DuckDuckGo	II - IV	-0.0272	0.0135	Inf	-0.0755	0.0212	-2.0107	>0.999	
Oregon	joe biden	DuckDuckGo	III - IV	-0.0894	0.0121	Inf	-0.1327	-0.0461	-7.3850	<0.001	***
Oregon	joe biden	Google	I - II	0.0187	0.0100	Inf	-0.0172	0.0545	1.8630	>0.999	
Oregon	joe biden	Google	I - III	0.0692	0.0098	Inf	0.0343	0.1041	7.0979	<0.001	***
Oregon	joe biden	Google	I - IV	0.0320	0.0122	Inf	-0.0116	0.0757	2.6263	>0.999	
Oregon	joe biden	Google	II - III	0.0506	0.0102	Inf	0.0139	0.0872	4.9388	<0.001	***
Oregon	joe biden	Google	II - IV	0.0134	0.0126	Inf	-0.0317	0.0585	1.0611	>0.999	
Oregon	joe biden	Google	III - IV	-0.0372	0.0120	Inf	-0.0800	0.0056	-3.1104	0.269	
Oregon	joe biden	Yahoo!	I - II	-0.0058	0.0095	Inf	-0.0397	0.0282	-0.6059	>0.999	
Oregon	joe biden	Yahoo!	I - III	0.0735	0.0095	Inf	0.0395	0.1076	7.7239	<0.001	***
Oregon	joe biden	Yahoo!	I - IV	0.0595	0.0106	Inf	0.0215	0.0976	5.5984	<0.001	***
Oregon	joe biden	Yahoo!	II - III	0.0793	0.0097	Inf	0.0444	0.1142	8.1392	<0.001	***
Oregon	joe biden	Yahoo!	II - IV	0.0653	0.0108	Inf	0.0265	0.1041	6.0269	<0.001	***
Oregon	joe biden	Yahoo!	III - IV	-0.0140	0.0099	Inf	-0.0495	0.0215	-1.4105	>0.999	

(8 in favour, 3 against, 24 in total)

Oregon - "us elections" query:

place	query	engine	period_pairwise	estimate	SE	df	asymp.LCL	asymp.UCL	z.ratio	p.value	sig
Oregon	us elections	Bing	I - II	-0.0427	0.0099	Inf	-0.0780	-0.0074	-4.3298	0.002	**
Oregon	us elections	Bing	I - III	0.1481	0.0085	Inf	0.1176	0.1787	17.3550	<0.001	***
Oregon	us elections	Bing	I - IV	0.2007	0.0092	Inf	0.1677	0.2336	21.8151	<0.001	***

Oregon	us elections	Bing	II - III	0.1908	0.0090	Inf	0.1588	0.2229	21.2988	<0.001	***
Oregon	us elections	Bing	II - IV	0.2434	0.0096	Inf	0.2090	0.2777	25.3679	<0.001	***
Oregon	us elections	Bing	III - IV	0.0525	0.0076	Inf	0.0253	0.0797	6.8981	<0.001	***
Oregon	us elections	DuckDuckGo	I - II	0.0251	0.0132	Inf	-0.0222	0.0724	1.8992	>0.999	
Oregon	us elections	DuckDuckGo	I - III	0.1926	0.0126	Inf	0.1476	0.2377	15.2953	<0.001	***
Oregon	us elections	DuckDuckGo	I - IV	0.1984	0.0132	Inf	0.1513	0.2454	15.0811	<0.001	***
Oregon	us elections	DuckDuckGo	II - III	0.1675	0.0132	Inf	0.1202	0.2148	12.6785	<0.001	***
Oregon	us elections	DuckDuckGo	II - IV	0.1733	0.0137	Inf	0.1241	0.2224	12.6063	<0.001	***
Oregon	us elections	DuckDuckGo	III - IV	0.0058	0.0126	Inf	-0.0395	0.0510	0.4562	>0.999	
Oregon	us elections	Google	I - II	0.0065	0.0103	Inf	-0.0302	0.0432	0.6325	>0.999	
Oregon	us elections	Google	I - III	0.0973	0.0098	Inf	0.0622	0.1323	9.9237	<0.001	***
Oregon	us elections	Google	I - IV	0.0903	0.0117	Inf	0.0483	0.1324	7.6875	<0.001	***
Oregon	us elections	Google	II - III	0.0908	0.0103	Inf	0.0539	0.1277	8.7974	<0.001	***
Oregon	us elections	Google	II - IV	0.0838	0.0122	Inf	0.0402	0.1275	6.8730	<0.001	***
Oregon	us elections	Google	III - IV	-0.0069	0.0114	Inf	-0.0476	0.0337	-0.6108	>0.999	
Oregon	us elections	Yahoo!	I - II	0.0101	0.0095	Inf	-0.0238	0.0439	1.0614	>0.999	
Oregon	us elections	Yahoo!	I - III	0.0338	0.0095	Inf	-0.0002	0.0678	3.5583	0.054	
Oregon	us elections	Yahoo!	I - IV	0.0407	0.0106	Inf	0.0027	0.0787	3.8341	0.018	*
Oregon	us elections	Yahoo!	II - III	0.0237	0.0097	Inf	-0.0110	0.0584	2.4477	>0.999	
Oregon	us elections	Yahoo!	II - IV	0.0306	0.0108	Inf	-0.0080	0.0693	2.8388	0.652	
Oregon	us elections	Yahoo!	III - IV	0.0069	0.0099	Inf	-0.0284	0.0423	0.6992	>0.999	

(13 in favour, 1 against, 24 in total)

Frankfurt - “donald trump” query:

Frankfurt	donald trump	Bing	I - II	-0.0498	0.0108	Inf	-0.0885	-0.0110	-4.5950	<0.001	***
Frankfurt	donald trump	Bing	I - III	0.0856	0.0093	Inf	0.0523	0.1188	9.2164	<0.001	***
Frankfurt	donald trump	Bing	I - IV	0.0870	0.0115	Inf	0.0458	0.1282	7.5553	<0.001	***
Frankfurt	donald trump	Bing	II - III	0.1353	0.0094	Inf	0.1018	0.1688	14.4549	<0.001	***
Frankfurt	donald trump	Bing	II - IV	0.1368	0.0116	Inf	0.0953	0.1782	11.8068	<0.001	***
Frankfurt	donald trump	Bing	III - IV	0.0014	0.0096	Inf	-0.0330	0.0359	0.1499	>0.999	
Frankfurt	donald trump	DuckDuckGo	I - II	0.0325	0.0137	Inf	-0.0165	0.0814	2.3731	>0.999	
Frankfurt	donald trump	DuckDuckGo	I - III	0.0322	0.0126	Inf	-0.0128	0.0771	2.5594	>0.999	
Frankfurt	donald trump	DuckDuckGo	I - IV	0.0455	0.0148	Inf	-0.0073	0.0984	3.0820	0.296	
Frankfurt	donald trump	DuckDuckGo	II - III	-0.0003	0.0141	Inf	-0.0509	0.0503	-0.0207	>0.999	
Frankfurt	donald trump	DuckDuckGo	II - IV	0.0131	0.0161	Inf	-0.0446	0.0708	0.8115	>0.999	
Frankfurt	donald trump	DuckDuckGo	III - IV	0.0134	0.0143	Inf	-0.0378	0.0646	0.9349	>0.999	
Frankfurt	donald trump	Google	I - II	-0.0768	0.0110	Inf	-0.1162	-0.0374	-6.9674	<0.001	***
Frankfurt	donald trump	Google	I - III	0.0150	0.0103	Inf	-0.0217	0.0517	1.4612	>0.999	
Frankfurt	donald trump	Google	I - IV	0.0520	0.0117	Inf	0.0101	0.0939	4.4357	0.001	**
Frankfurt	donald trump	Google	II - III	0.0918	0.0107	Inf	0.0535	0.1301	8.5676	<0.001	***
Frankfurt	donald trump	Google	II - IV	0.1287	0.0121	Inf	0.0854	0.1721	10.6218	<0.001	***
Frankfurt	donald trump	Google	III - IV	0.0370	0.0110	Inf	-0.0024	0.0763	3.3631	0.111	
Frankfurt	donald trump	Yahoo!	I - II	0.0092	0.0112	Inf	-0.0308	0.0492	0.8222	>0.999	
Frankfurt	donald trump	Yahoo!	I - III	0.0641	0.0095	Inf	0.0302	0.0980	6.7713	<0.001	***
Frankfurt	donald trump	Yahoo!	I - IV	0.0691	0.0101	Inf	0.0331	0.1052	6.8590	<0.001	***
Frankfurt	donald trump	Yahoo!	II - III	0.0549	0.0097	Inf	0.0201	0.0897	5.6385	<0.001	***
Frankfurt	donald trump	Yahoo!	II - IV	0.0599	0.0103	Inf	0.0230	0.0969	5.7999	<0.001	***
Frankfurt	donald trump	Yahoo!	III - IV	0.0050	0.0076	Inf	-0.0223	0.0323	0.6595	>0.999	

(11 in favour, 2 against, 24 in total)

Frankfurt - “joe biden” query:

Frankfurt	joe biden	Bing	I - II	-0.0177	0.0108	Inf	-0.0563	0.0208	-1.6435	>0.999	
Frankfurt	joe biden	Bing	I - III	0.0893	0.0093	Inf	0.0561	0.1225	9.6268	<0.001	***
Frankfurt	joe biden	Bing	I - IV	-0.0598	0.0115	Inf	-0.1009	-0.0186	-5.1930	<0.001	***
Frankfurt	joe biden	Bing	II - III	0.1070	0.0093	Inf	0.0737	0.1403	11.4989	<0.001	***
Frankfurt	joe biden	Bing	II - IV	-0.0421	0.0115	Inf	-0.0833	-0.0008	-3.6446	0.039	*
Frankfurt	joe biden	Bing	III - IV	-0.1491	0.0096	Inf	-0.1836	-0.1146	-15.4662	<0.001	***
Frankfurt	joe biden	DuckDuckGo	I - II	0.0425	0.0135	Inf	-0.0059	0.0908	3.1437	0.240	
Frankfurt	joe biden	DuckDuckGo	I - III	0.1101	0.0118	Inf	0.0678	0.1524	9.3080	<0.001	***
Frankfurt	joe biden	DuckDuckGo	I - IV	-0.0064	0.0141	Inf	-0.0569	0.0441	-0.4531	>0.999	
Frankfurt	joe biden	DuckDuckGo	II - III	0.0676	0.0133	Inf	0.0201	0.1151	5.0929	<0.001	***
Frankfurt	joe biden	DuckDuckGo	II - IV	-0.0489	0.0153	Inf	-0.1038	0.0060	-3.1862	0.208	
Frankfurt	joe biden	DuckDuckGo	III - IV	-0.1165	0.0130	Inf	-0.1628	-0.0702	-8.9942	<0.001	***
Frankfurt	joe biden	Google	I - II	-0.0517	0.0108	Inf	-0.0903	-0.0131	-4.7931	<0.001	***
Frankfurt	joe biden	Google	I - III	0.0304	0.0101	Inf	-0.0059	0.0666	2.9981	0.391	
Frankfurt	joe biden	Google	I - IV	0.0136	0.0116	Inf	-0.0277	0.0549	1.1782	>0.999	
Frankfurt	joe biden	Google	II - III	0.0821	0.0104	Inf	0.0448	0.1194	7.8776	<0.001	***
Frankfurt	joe biden	Google	II - IV	0.0653	0.0118	Inf	0.0231	0.1076	5.5353	<0.001	***
Frankfurt	joe biden	Google	III - IV	-0.0168	0.0107	Inf	-0.0552	0.0217	-1.5600	>0.999	
Frankfurt	joe biden	Yahoo!	I - II	-0.0125	0.0111	Inf	-0.0523	0.0272	-1.1297	>0.999	
Frankfurt	joe biden	Yahoo!	I - III	0.0748	0.0095	Inf	0.0410	0.1087	7.9037	<0.001	***
Frankfurt	joe biden	Yahoo!	I - IV	0.0618	0.0101	Inf	0.0257	0.0978	6.1307	<0.001	***
Frankfurt	joe biden	Yahoo!	II - III	0.0874	0.0096	Inf	0.0529	0.1218	9.0700	<0.001	***
Frankfurt	joe biden	Yahoo!	II - IV	0.0743	0.0102	Inf	0.0377	0.1109	7.2630	<0.001	***
Frankfurt	joe biden	Yahoo!	III - IV	-0.0130	0.0076	Inf	-0.0403	0.0143	-1.7079	>0.999	

(10 in favour, 4 against, 24 in total)

Frankfurt - “us elections” query:

Frankfurt	us elections	Bing	I - II	0.0215	0.0107	Inf	-0.0167	0.0597	2.0122	>0.999	
Frankfurt	us elections	Bing	I - III	0.0561	0.0093	Inf	0.0230	0.0892	6.0584	<0.001	***
Frankfurt	us elections	Bing	I - IV	0.0812	0.0114	Inf	0.0402	0.1221	7.0895	<0.001	***
Frankfurt	us elections	Bing	II - III	0.0346	0.0092	Inf	0.0016	0.0676	3.7537	0.025	*
Frankfurt	us elections	Bing	II - IV	0.0597	0.0114	Inf	0.0188	0.1005	5.2242	<0.001	***

Frankfurt	us elections	Bing	III - IV	0.0251	0.0096	Inf	-0.0092	0.0593	2.6153	>0.999	
Frankfurt	us elections	DuckDuckGo	I - II	0.0270	0.0142	Inf	-0.0239	0.0780	1.8989	>0.999	
Frankfurt	us elections	DuckDuckGo	I - III	0.1264	0.0136	Inf	0.0777	0.1752	9.2801	<0.001	***
Frankfurt	us elections	DuckDuckGo	I - IV	0.1023	0.0141	Inf	0.0519	0.1526	7.2669	<0.001	***
Frankfurt	us elections	DuckDuckGo	II - III	0.0994	0.0147	Inf	0.0468	0.1520	6.7614	<0.001	***
Frankfurt	us elections	DuckDuckGo	II - IV	0.0753	0.0151	Inf	0.0212	0.1293	4.9773	<0.001	***
Frankfurt	us elections	DuckDuckGo	III - IV	-0.0241	0.0136	Inf	-0.0728	0.0246	-1.7729	>0.999	
Frankfurt	us elections	Google	I - II	0.0264	0.0110	Inf	-0.0130	0.0659	2.3974	>0.999	
Frankfurt	us elections	Google	I - III	0.0764	0.0104	Inf	0.0392	0.1136	7.3541	<0.001	***
Frankfurt	us elections	Google	I - IV	0.1149	0.0118	Inf	0.0726	0.1573	9.7095	<0.001	***
Frankfurt	us elections	Google	II - III	0.0500	0.0109	Inf	0.0112	0.0888	4.6067	<0.001	***
Frankfurt	us elections	Google	II - IV	0.0885	0.0122	Inf	0.0448	0.1323	7.2378	<0.001	***
Frankfurt	us elections	Google	III - IV	0.0385	0.0112	Inf	-0.0016	0.0787	3.4326	0.086	
Frankfurt	us elections	Yahoo!	I - II	0.0129	0.0111	Inf	-0.0266	0.0525	1.1693	>0.999	
Frankfurt	us elections	Yahoo!	I - III	0.0354	0.0095	Inf	0.0015	0.0693	3.7393	0.027	*
Frankfurt	us elections	Yahoo!	I - IV	0.0405	0.0101	Inf	0.0045	0.0766	4.0279	0.008	**
Frankfurt	us elections	Yahoo!	II - III	0.0225	0.0096	Inf	-0.0119	0.0568	2.3393	>0.999	
Frankfurt	us elections	Yahoo!	II - IV	0.0276	0.0102	Inf	-0.0089	0.0641	2.7087	0.973	

(12 in favour, 0 against, 24 in total)

Degrees-of-freedom method: asymptotic
Confidence level used: 0.95
Conf-level adjustment: bonferroni method for 144 estimates
P value adjustment: bonferroni method for 144 tests

H2e & H2f - Contrasts Table [emmeans(the_model, ~ query|(engine:period), lmer.df = "asymptotic", at = list(place = c("Oregon"), query=c("donald trump", "joe biden")))]:

place	engine	period	query_pairwise	estimate	SE	df	asymp.LCL	asymp.UCL	z.ratio	p.value	sig
**H2e - Period I to III (Oregon):**											
Oregon	Bing	I	donald trump - joe biden	0.2199	0.0062	Inf	0.2004	0.2394	35.6777	<0.001	***
Oregon	Bing	II	donald trump - joe biden	0.2007	0.0072	Inf	0.1779	0.2234	27.9051	<0.001	***
Oregon	Bing	III	donald trump - joe biden	0.1433	0.0061	Inf	0.1241	0.1625	23.6115	<0.001	***
Oregon	DuckDuckGo	I	donald trump - joe biden	0.0633	0.0086	Inf	0.0361	0.0906	7.3440	<0.001	***
Oregon	DuckDuckGo	II	donald trump - joe biden	0.0905	0.0110	Inf	0.0558	0.1251	8.2543	<0.001	***
Oregon	DuckDuckGo	III	donald trump - joe biden	0.1274	0.0104	Inf	0.0944	0.1604	12.2057	<0.001	***
Oregon	Google	I	donald trump - joe biden	-0.0391	0.0060	Inf	-0.0581	-0.0201	-6.5014	<0.001	***
Oregon	Google	II	donald trump - joe biden	-0.0206	0.0076	Inf	-0.0447	0.0036	-2.6945	0.226	
Oregon	Google	III	donald trump - joe biden	-0.0163	0.0085	Inf	-0.0432	0.0107	-1.9092	>0.999	
Oregon	Yahoo!	I	donald trump - joe biden	-0.0176	0.0055	Inf	-0.0351	0.0000	-3.1715	0.049	*
Oregon	Yahoo!	II	donald trump - joe biden	-0.0302	0.0065	Inf	-0.0506	-0.0098	-4.6788	<0.001	***
Oregon	Yahoo!	III	donald trump - joe biden	-0.0049	0.0078	Inf	-0.0295	0.0197	-0.6307	>0.999	
**H2e - Period I to III (Frankfurt):**											
Frankfurt	Bing	I	donald trump - joe biden	0.0028	0.0076	Inf	-0.0211	0.0268	0.3721	>0.999	
Frankfurt	Bing	II	donald trump - joe biden	0.0349	0.0081	Inf	0.0092	0.0606	4.2932	<0.001	***
Frankfurt	Bing	III	donald trump - joe biden	0.0066	0.0056	Inf	-0.0112	0.0243	1.1724	>0.999	
Frankfurt	DuckDuckGo	I	donald trump - joe biden	0.0335	0.0094	Inf	0.0039	0.0631	3.5736	0.011	*
Frankfurt	DuckDuckGo	II	donald trump - joe biden	0.0435	0.0131	Inf	0.0021	0.0849	3.3243	0.028	*
Frankfurt	DuckDuckGo	III	donald trump - joe biden	0.1114	0.0106	Inf	0.0780	0.1448	10.5486	<0.001	***
Frankfurt	Google	I	donald trump - joe biden	-0.0167	0.0073	Inf	-0.0399	0.0065	-2.2747	0.733	
Frankfurt	Google	II	donald trump - joe biden	0.0084	0.0086	Inf	-0.0187	0.0354	0.9781	>0.999	
Frankfurt	Google	III	donald trump - joe biden	-0.0013	0.0083	Inf	-0.0276	0.0250	-0.1558	>0.999	
Frankfurt	Yahoo!	I	donald trump - joe biden	-0.0199	0.0078	Inf	-0.0446	0.0049	-2.5401	0.355	
Frankfurt	Yahoo!	II	donald trump - joe biden	-0.0416	0.0087	Inf	-0.0692	-0.0140	-4.7702	<0.001	***
Frankfurt	Yahoo!	III	donald trump - joe biden	-0.0092	0.0056	Inf	-0.0269	0.0086	-1.6341	>0.999	
**H2f - Period IV (Oregon):**											
Oregon	Bing	IV	donald trump - joe biden	0.0529	0.0078	Inf	0.0282	0.0777	6.7659	<0.001	***
Oregon	DuckDuckGo	IV	donald trump - joe biden	0.0196	0.0133	Inf	-0.0223	0.0615	1.4783	>0.999	
Oregon	Google	IV	donald trump - joe biden	-0.0443	0.0129	Inf	-0.0851	-0.0036	-3.4446	0.018	*
Oregon	Yahoo!	IV	donald trump - joe biden	-0.0230	0.0098	Inf	-0.0539	0.0080	-2.3481	0.604	
**H2f - Period IV (Frankfurt):**											
Frankfurt	Bing	IV	donald trump - joe biden	-0.1440	0.0105	Inf	-0.1771	-0.1108	-13.7277	<0.001	***
Frankfurt	DuckDuckGo	IV	donald trump - joe biden	-0.0184	0.0149	Inf	-0.0654	0.0285	-1.2418	>0.999	
Frankfurt	Google	IV	donald trump - joe biden	-0.0550	0.0111	Inf	-0.0903	-0.0198	-4.9424	<0.001	***
Frankfurt	Yahoo!	IV	donald trump - joe biden	-0.0272	0.0069	Inf	-0.0491	-0.0053	-3.9364	0.003	**

Degrees-of-freedom method: asymptotic
Confidence level used: 0.95
Conf-level adjustment: bonferroni method for 32 estimates
P value adjustment: bonferroni method for 32 tests

Table S6. Linear mixed model, ANOVA and contrasts table for the novelty of the US-related queries. The top row of the table presents the linear mixed model, the general resulting statistics and random effect statistics. The fixed effects combinations are omitted and instead the ANOVA Table (middle row) is presented. The third row presents the region contrasts within the query-engine pair (across periods) to test H2a. The fourth row presents the engine contrasts within queries to test H2b; only the contrasts of Oregon are

presented, and only the contrasts between two subsequent engines ordered by their average novelty which is presented at the end of the row. The fifth row presents the period contrasts within the query-engine pair to test H2c; only including the contrasts for Oregon. The sixth row presents the query contrasts within period-engine to test H2d and H2e; only Oregon contrasts are presented. Contrasts are highlighted green if they support the hypothesis, yellow if they discard the hypothesis, and red if the effect is in the opposite direction of the predicted one.

Appendix S7. Unique items for US elections query for Periods I, II and II

	Oregon				Frankfurt			
	Bing	DDG	Google	Yahoo!	Bing	DDG	Google	Yahoo!
donald trump	3599	2436	1332	719	1913	1767	1161	744
joe biden	1110	1225	1557	852	1870	1001	1154	932
us elections	4013	2126	1744	364	533	1411	1442	357
Trump to Biden	3.24	1.99	0.86	0.84	1.02	1.77	1.01	0.8
Biden to Trump	0.31	0.5	1.17	1.18	0.98	0.57	0.99	1.25

Table S7. Unique news items for each election query in periods I, II and III. The table shows the number of unique news items for each query, as well as the proportions between que query corresponding to the candidate. The first row indicates the region and the second row the search engine. The following three rows indicate the number of unique links for each of the queries, and the last two the proportion of unique results of “donald trump” to “joe biden”, and its inverse, “joe biden” to “donald trump”.

Appendix S8. Novelty of search results over time in Frankfurt.

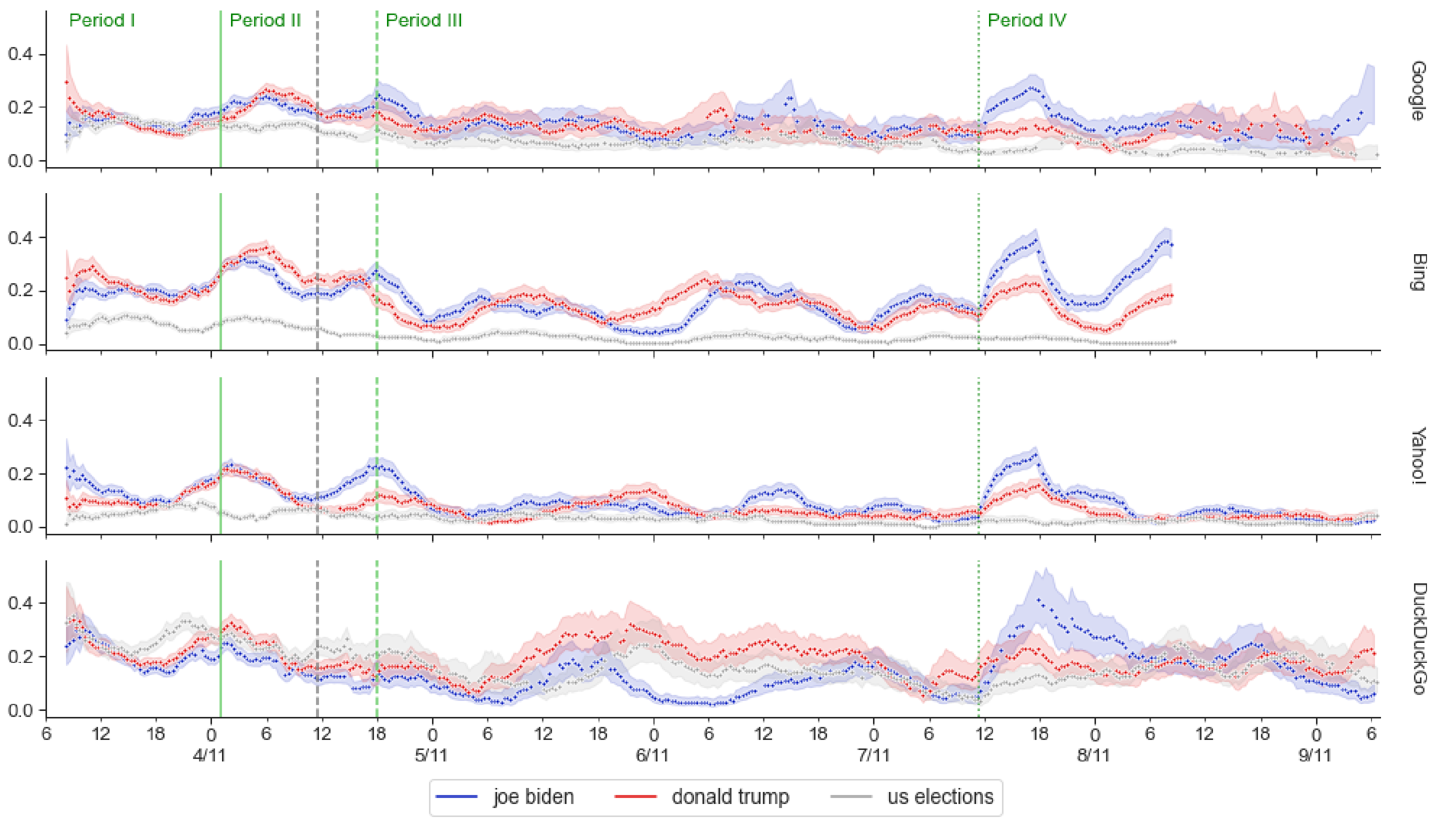

Figure S8. Novelty of search results over time in Frankfurt. The four plots present the rolled average novelty (of the last 6h, n=18) for each search engine (right label). The X-axis shows the day (major ticks) and hour (minor ticks) of the round in which the novelty was measured. The Y-axis shows the novelty truncated to .4 (maximum theoretical value 1.0). Each trace represents each of the query terms indicated on the legend. The green vertical lines divide each plot in four periods indicated in the label at the top. The grey dotted vertical line in Period II indicates the transition between collection A and B. Only the results collected in Frankfurt are shown. The bands indicate bootstrapped 95% confidence intervals.
